# CENP-B protects centromere chromatin integrity by facilitating histone deposition via the H3.3-specific chaperone Daxx

**DOI:** 10.1186/s13072-017-0164-y

**Published:** 2017-12-22

**Authors:** Viacheslav M. Morozov, Serena Giovinazzi, Alexander M. Ishov

**Affiliations:** 1 Department of Anatomy and Cell Biology, University of Florida College of Medicine, and University of Florida Cancer Center, 2033 Mowry Road, Room 358, Gainesville, FL 32610 USA; 2 Present Address: Division of Food Safety, Florida Department of Agriculture and Consumer Services, Tallahassee, FL USA

**Keywords:** Histone chaperone Daxx, H3.3, CENP-B, Centromere, SUMO, Chromatin, SIM

## Abstract

**Background:**

The main chromatin unit, the nucleosome, can be modulated by the incorporation of histone variants that, in combination with posttranslational histones modifications, determine epigenetics properties of chromatin. Understanding the mechanism that creates a histone variants landscape at different genomic elements is expected to elevate our comprehension of chromatin assembly and function. The Daxx chaperone deposits transcription-associated histone H3.3 at centromeres, but mechanism of centromere-specific Daxx targeting remains unclear.

**Results:**

In this study, we identified an unexpected function of the constitutive centromeric protein CENP-B that serves as a “beacon” for H3.3 incorporation. CENP-B depletion reduces Daxx association and H3.3 incorporation at centromeres. Daxx/CENP-B interaction and Daxx centromeric association are SUMO dependent and requires SIMs of Daxx. Depletion of SUMO-2, but not SUMO-1, decreases Daxx/CENP-B interaction and reduces centromeric accumulation of Daxx and H3.3, demonstrating distinct functions of SUMO paralogs in H3.3 chaperoning. Finally, disruption of CENP-B/Daxx-dependent H3.3 pathway deregulates heterochromatin marks H3K9me3, ATRX and HP1α at centromeres and elevates chromosome instability.

**Conclusion:**

The demonstrated roles of CENP-B and SUMO-2 in H3.3 loading reveal a novel mechanism controlling chromatin maintenance and genome stability. Given that CENP-B is the only centromere protein that binds centromere-specific DNA elements, our study provides a new link between centromere DNA and unique epigenetic landscape of centromere chromatin.

**Electronic supplementary material:**

The online version of this article (10.1186/s13072-017-0164-y) contains supplementary material, which is available to authorized users.

## Background

In eukaryotic cells, chromatin regulates essential nuclear processes, such as transcription, DNA replication/repair, RNA processing, stability and export. The main chromatin unit is the nucleosome; it can be modulated by posttranslational histones modifications and also by the incorporation of histone variants [1] that, in combination, determine epigenetic properties of chromatin. The “H3 barcode” hypothesis [2] is based on differences between histone H3 variants, H3.1/3.2 and H3.3, and postulates that distinct incorporation of H3 variants creates specialized functional chromatin territories. Therefore, understanding the mechanism that creates a histone variants landscape at different genomic elements will elevate our comprehension of chromatin assembly and function.

Despite high similarity between the canonical histone H3.1/3.2 and H3.3 amino acid sequences, their biological properties, as well as their chaperones, are different [3–5]. The Daxx/ATRX chaperone complex loads H3.3 into repetitive genome elements, such as telomeres [6] and centromeres [6, 7]. It was shown that the chromatin-associated protein DEK regulates Daxx/ATRX-dependent incorporation of H3.3 at telomeres [8]; however, how the Daxx-containing complex is targeted to centromeres to guarantee the spatial positioning and functional specificity of this chaperone remains unclear [9].

The centromere is a specialized chromosome region that serves as a platform for the formation of the kinetochore, a multi-protein complex essential for the microtubule attachment necessary for proper chromosome segregation during cell division (reviewed recently in [10, 11]). The centromere is composed of several conservative proteins loaded on arrays of tandem DNA repeats (TR). It was suggested that both genetic (DNA repeats) and epigenetic (centromere proteins and histone modification) components participate in centromere functional integrity and maintenance. Yet, DNA repeats have high interspecies variability and are neither sufficient nor necessary for centromere functions [12]. Thus, despite the absence of centromere repeats, a neocentromere can serve as an inheritable platform for kinetochore assembly at ectopic loci (review in [13]), emphasizing the role of epigenetic components in the maintenance of a functional centromere.

In most organisms, centromere chromatin can be divided into two regions: the centromere core that is enriched with the centromere-specific H3 variant, CENP-A [14] and H3K4me2 [15]), and pericentromere, usually associated with heterochromatic epigenetic marks as H3K9me2/3 [15]; it was suggested that the epigenetic modifications at these two regions of chromatin exist in dynamic balance [16].

At the DNA level, human centromeres are composed of tandem arrays of α-satellite repeats [17] organized as higher-order repeat units (HORs [17]) flanked by divergent monomers with no detectable higher-order structure. HORs α-satellites are enriched with 17-bp motif, CENP-B box, which is recognized by the major constitutive centromere protein, centromere protein B (CENP-B). In addition to HORs, α-satellite dimers were recently found dominating human centromeres [18]; these dimers are flanked by CENP-B boxes and are associated with CENP-B [18]. Centromeres contain alternating blocks of CENP-A and H3 nucleosomes, which are in physically distinct locations in 3-D structure; at mitotic chromosomes, CENP-A is mostly associated with the kinetochores and H3 is associated with the “inner centromere” region between the kinetochores [19, 20].

CENP-B is the only centromere protein that binds a specific DNA sequence, CENP-B box [21], and is highly conserved among mammalian species, suggesting that CENP-B is required for an essential cell function. Therefore, CENP-B may represent a structural and functional link between the centromeric DNA and the kinetochore, shaping the proper landscape for centromere formation and borders. Thus, CENP-B binding to CENP-B boxes affects positioning and loading of CENP-A on alphoid DNA [22, 23] potentially via recruitment of CENP-C to the centromere [24, 25]. CENP-B regulates CENP-A chromatin assembly and maintenance of H3K9 methylation [23]; in addition, a series of studies demonstrated the necessity of CENP-B and CENP-B boxes in chromatin formation at human artificial chromosome (HAC) and their propagation in cells [26]. Recent studies also uncovered the functions of CENP-B in centromere DNA replication [27] and CENP-C recruitment [28]. Interestingly, CENP-B is found in the kinetochore and in the inner centromere [29–31]; thus, it is associated with both CENP-A and H3-reach areas of centromere and is presumably functional at both locations.

In the current study, we identified a novel and unexpected function of CENP-B in the deposition of H3.3 at human centromeres by the chaperone Daxx. This CENP-B function is facilitated by SUMO-dependent recruitment of Daxx chaperone complex for the incorporation of H3.3 into CENP-B box-containing centromere repeats. Disruption of CENP-B/Daxx-dependent H3.3 loading by CENP-B or Daxx knockout drastically reduces heterochromatic marks and elevates chromosome instability, suggesting a new CENP-B-dependent mechanism that controls chromatin landscape and genome integrity.

## Results

### Daxx is associated with centromeric protein CENP-B

**Fig. 1:**
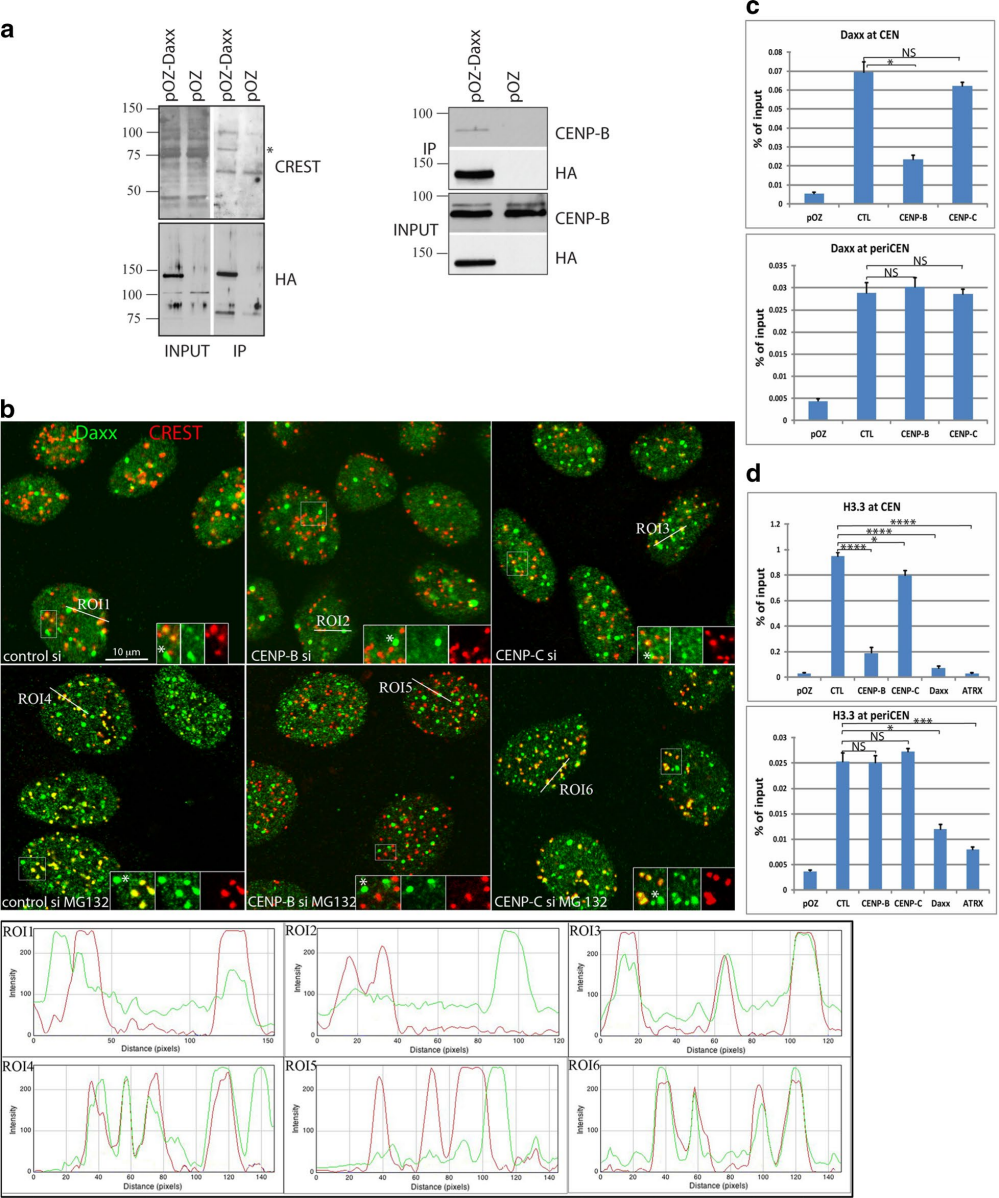
CENP-B interacts with Daxx and targets Daxx to centromeres for H3.3 loading. **a** FLAG IP from chromatin fraction of HEp2 cells expressing FLAG-HA-Daxx probed with CREST antibodies (left). Major band ~ 80 kDa was identified as CENP-B with specific antibodies (right). **b** Depletion of CENP-B prevents Daxx accumulation at centromeres. Representative images of HEp2 cells transfected with scrambled siRNA (left), CENP-B siRNA (middle) and CENP-C siRNA (right). Daxx (green) is associated with subpopulation of centromeres (CREST, red) in HEp2 cells (top row); treatment with MG132 (bottom row) increases number of PML bodies [36] and boosts association of Daxx with centromeres [36]. Depletion of CENP-B, but not CENP-C, reduced association of Daxx with centromeres in both tested conditions. PML nuclear bodies are marked with asterisks in the inserts. Quantitation of colocalization is presented in Additional file 3: Fig. S3. Graphs in the bottom panel show fluorescence intensity of Daxx (green) and CREST (red) signal along the region of interest (ROI). **c** Daxx association with α-satellite (CEN) and HS3 satellite (periCEN) were tested by ChIP assay. CENP-B depletion abolished Daxx association with CEN (CENP-B box positive α-satellite), while did not affect periCEN association (CENP-B box negative HS3). **d** ChIP analysis of H3.3 enrichment at CEN and periCEN in HEp2 cells expressing FLAG-HA-H3.3. Depletion of CENP-B reduced H3.3 enrichment at CEN, but not at periCEN. Depletion of Daxx and ATRX reduced association of H3.3 with both CEN and periCEN, confirming functionality of Daxx/ATRX complex as a histone chaperone at both genomic elements. Bars represent the mean ± SD (*n* = 3). Statistical analysis was performed by one-way ANOVA followed by Tukey’s multiple comparisons test. ****P < 0.0001; ***P < 0.001; **P < 0. 01; *P < 0.1; NS, nonsignificant

**Fig. 2:**
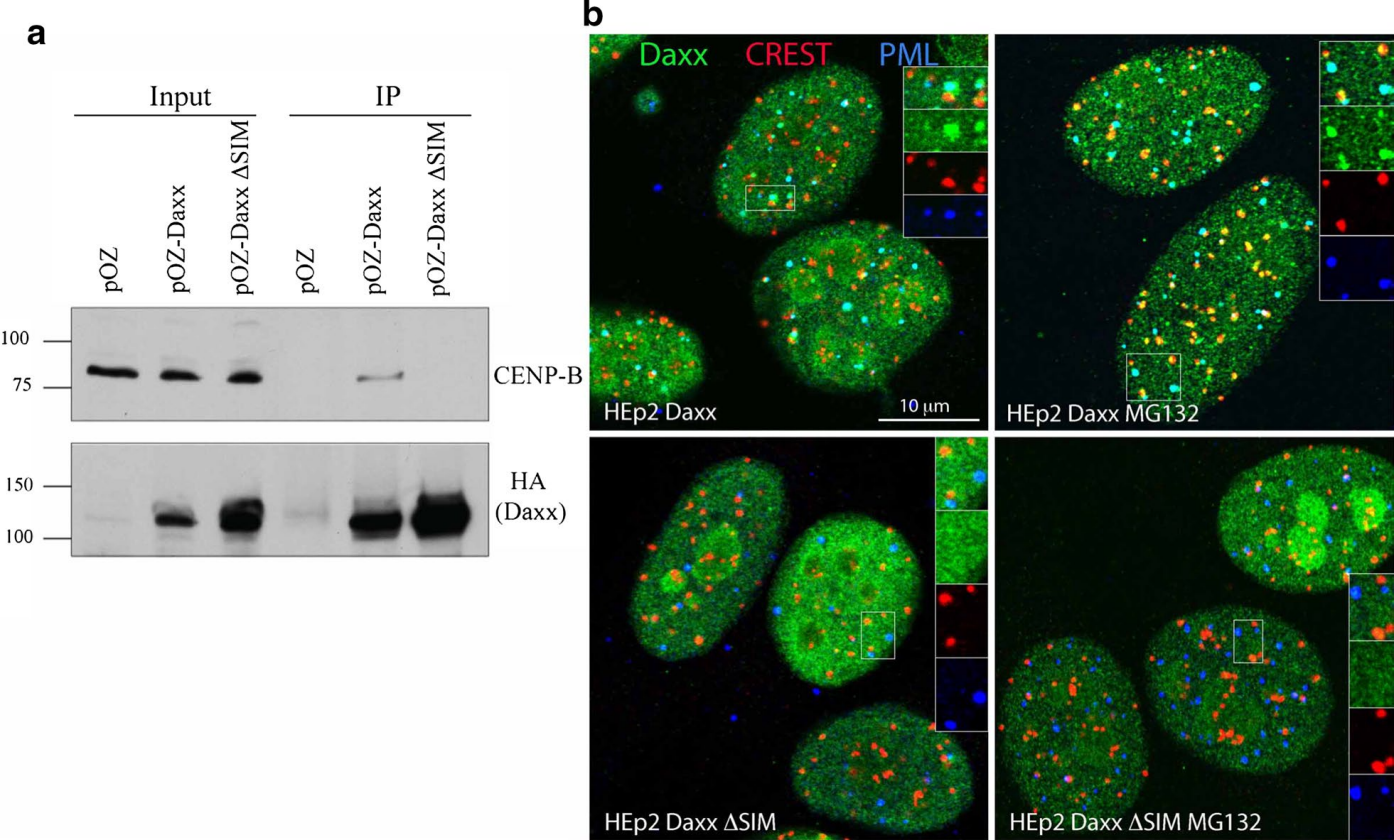
SUMO interaction domains (SIMs) of Daxx are required for CENP-B interaction and centromeres targeting. **a** Daxx SIMs are required for interaction with CENP-B. Western blot analysis of FLAG IP from HEp2 cells expressing FLAG-HA-Daxx and FLAG-HA-DaxxΔSIM probed with anti-CENP-B ab’s. CENP-B co-IP with DaxxWT, but not with DaxxΔSIM. pOZ: negative control. **b** Daxx SIMs are required for Daxx accumulation at centromeres. Representative images of HEp2 cells expressing FLAG-HA-DaxxWT (top) and FLAG-HA-DaxxΔSIM (bottom) are shown. In untreated cells (left), DaxxWT (anti-HA, green) co-localizes with PML (blue) and some centromeres (CREST, red); the latter association is increased after MG132 treatment (right). DaxxΔSIM is not associated with either ND10/PML bodies or centromeres in untreated as well as MG132 treated cells

**Fig. 3:**
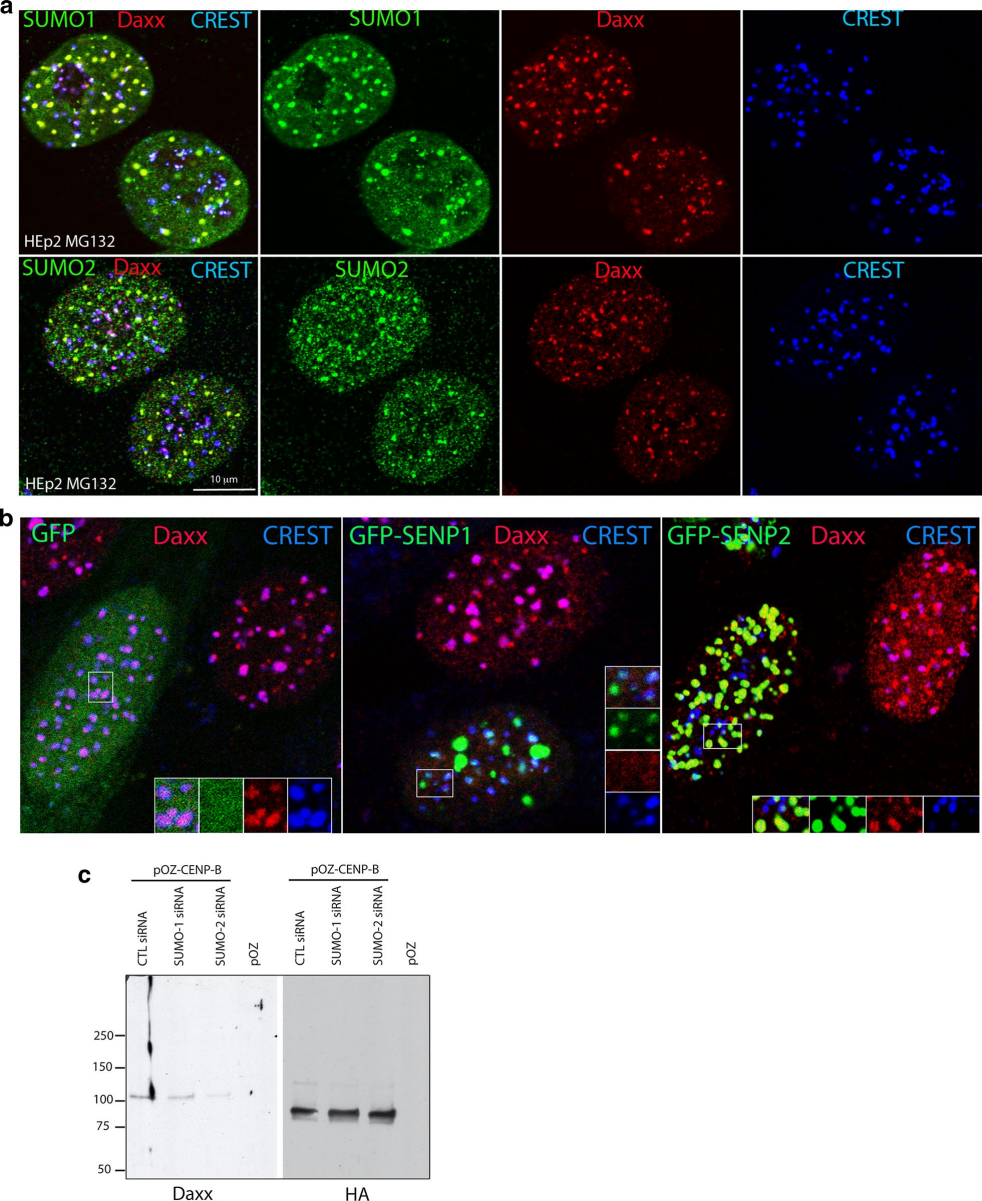
SUMO pathway is required for Daxx recruitment to centromeres. **a** Both SUMO-1 and SUMO-2 are accumulated with Daxx at centromeres. SUMO-1 (green, upper row) and SUMO-2 (green, bottom row) co-localize with Daxx (red) at centromeres (CREST, blue) in HEp2 cells after treatment with proteasome inhibitor MG132. **b** Expression of SUMO de-conjugation enzymes SENPs affects Daxx accumulation at centromeres. Representative images of HEp2 cells transiently expressing GFP (left), GFP-SENP1 (middle) or GFP-SENP2 (right) and treated with MG132, 4 h. SENP1-depleted Daxx (red) from centromeres (CREST, blue) and dispersed Daxx homogenously in nucleoplasm; SENP2-depleted Daxx from centromeres and co-localized with Daxx in ND10-like domains. **c** Daxx interaction with CENP-B is SUMO-2 dependent. FLAG-HA-CENP-B expressing HEp2 cells were transfected with scrambled siRNA (CTL), SUMO-1 or SUMO-2 siRNA (Additional file 4: Fig. S4, top for the levels of SUMO-1 and SUMO-2 depletion) and treated with MG132. Chromatin fraction of cell lysates (Additional file 4: Fig. S4, bottom for Daxx input) was IP with anti-FLAG-M2 magnetic beads. Eluates were immunoblotted with HA (for CENP-B) and Daxx. Relative to control siRNA, SUMO-2 depletion reduced Daxx co-IP with CENP-B to 0.2, while depletion of SUMO-1 had minor effect reducing Daxx pull-down to 0.7

Daxx/ATRX complex deposits H3.3 histone variant at centromeres [6, 7, 32]; however, it is still unclear how Daxx complex is targeted to these genomic loci to execute this function. To understand the mechanism of Daxx recruitment to centromeres, we searched for chromatin-associated Daxx interaction partner(s) at centromere. We immunoprecipitated (IP) Daxx from the chromatin-associated fraction of human laryngeal carcinoma HEp2 cells stably expressing FLAG-HA-Daxx. The co-purified proteins were probed with CREST human autoimmune antibody that recognizes several constitutive centromere proteins [33]. CREST antibody identified a single specific polypeptide of ~ 80 kDa in Daxx chromatin-associated IP (Fig. 1a, left). Based on the polypeptide size, we reasoned that the identified protein can be the major constitutive centromere protein CENP-B, and immunobloting with antibodies specific to CENP-B confirmed our assumption (Fig. 1a, right). CENP-B signal was faint but well reproducible (see also Fig. 2a and Additional file 1: Fig. S1A), that can be explained by the relatively small population of cells (about 10%) that exhibit Daxx association at centromeres (see Fig. 1b and Ref. [32]). Reciprocal IP validated Daxx association with CENP-B (Additional file 1: Fig. S1B, see also Fig. 3c). We concluded that chromatin-associated fraction of Daxx cellular milieu was associated with CENP-B; yet, Co-IP experiments do not allow distinguishing between direct and indirect (as components of the same complex) interaction between these proteins.

### CENP-B is required for accumulation of Daxx at centromeres

Daxx was previously found localized at PML nuclear bodies/ND10 [34], heterochromatin [35], and also at centromeres in a subpopulation of cells [32, 36]; our data suggest that CENP-B can target Daxx to centromeres. Thus, we tested next whether the newly identified association with CENP-B is required for the localization of Daxx at centromeres. Daxx interaction with another constitutive centromere protein, CENP-C, was previously demonstrated by the yeast interaction assay [37]; thus, we also tested whether CENP-C is involved in Daxx targeting at centromeres. We found that Daxx was associated with centromeres in about 10% of cells, confirming our previous observations [32]. Depletion of CENP-B (see Additional file 2: Fig. S2 for CENP-B and CENP-C depletion efficiency) abolished co-localization of Daxx and centromeres (Fig. 1b, top middle: CENP-B siRNA). Daxx association with centromeres was not affected after depletion of CENP-C (Fig. 1b, top left: scramble siRNA; top right: CENP-C siRNA). Proteasome inhibitor MG132 boosts Daxx accumulation at centromeres [36] (Fig. 1b, bottom row, left), potentially via elevated sumoylation (see Figs. 1b, 2b, Ref. [38]). In these conditions, depletion of CENP-B, but not CENP-C, prevented Daxx accumulation at centromeres similarly to control conditions (Fig. 1b, bottom row, center and right, and quantitation Additional file 3: Fig. S3).

Next, we performed ChIP assay to confirm and to quantify CENP-B contribution in Daxx association with centromere, specifically with CENP-B box positive α-satellite repeats [21, 39]. We used set of primers to α-satellite repeats [40] that we previously characterized as specific to all human centromeres by in situ hybridization in mitotic and interphase cells [32]. Depletion of CENP-B substantially reduced Daxx association with α-satellite repeats (thereafter referred to as “CEN” in ChIP experiments), while transfection of CENP-C siRNA had minor effect (Fig. 1c, top). Pericentromere (periCEN) regions in humans consist of arrays of satellite repeats, including CENP-B box negative Human Satellite DNA 3 (HS3). HS3 arrays of different size are located at pericentromere regions in most human chromosomes [41]. We used a set of primers specific to HS3 of chromosome 9 (HS3–9). Specificity of primers was characterized by the in situ hybridization in mitotic and interphase cells [32]. As expected, enrichment of Daxx at CENP-B box negative HS3–9 repeats (hereafter refer as “periCEN” in ChIP experiments) was not changed after depletion of either CENP-B or CENP-C (Fig. 1c, bottom). Combination of IF and ChIP results allowed us to conclude that Daxx association with centromeres, specifically with the CENP-B box positive α-satellite repeats, is CENP-B dependent.

### CENP-B is required for H3.3 association with centromeres

Since CENP-B is required for the localization of H3.3 chaperone Daxx at the major centromere components, α-satellite repeats, it is reasonable to expect that CENP-B also mediates Daxx-dependent loading of H3.3 at these genomic elements. We tested this possibility by ChIP on HEp2 cells expressing FLAG-HA-H3.3 (for cell line characterization, see [32]). Depletion of CENP-B reduced H3.3 enrichment at CEN, but not at CENP-B box negative periCEN (Fig. 1d). Depletion of CENP-C reduces H3.3 level at CEN; yet, this effect is minor comparatively to the depletion of either Daxx or CENP-B and can be explained by recently observed inter-dependence between CENP-B and CENP-C [28]. Depletion of either component of Daxx chaperone complex, Daxx or ATRX, reduced levels of H3.3 at CEN and also at periCEN (Fig. 1d), confirming Daxx/ATRX-dependent incorporation of H3.3 at both genome elements. Collectively, these data suggest that Daxx chaperone complex is targeted to specific loci (CENP-B box positive α-satellite and CENP-B box negative pericentromeric repeats HS3) by different mechanisms and indicate that loading, maintenance or turnover of histone variant H3.3 at the major component of centromeres, α-satellite repeats, is CENP-B dependent.

### SUMO interaction motifs (SIMs) of Daxx are required for the association with CENP-B and accumulation at centromeres

Daxx interaction with a number of proteins is mediated by sumoylation (modification by SUMO) of these interaction partners (review in [42]); moreover, Daxx intra-nuclear targeting to the ND10/PML bodies requires PML sumoylation [34, 43]. Hence, we tested whether sumoylation can also regulate recruitment of Daxx to centromeres. Daxx has two SUMO interaction motifs (SIMs) that are required for its interaction with sumoylated partners [44, 45]. First, we tested whether Daxx SIMs are required for CENP-B association by co-IP, comparing Daxx wild type (DaxxWT) and a Daxx mutant with both SIMs inactivated (DaxxΔSIM, [45]) in HEp2 cells stably expressing DaxxWT or DaxxΔSIM. Results indicated that mutations of SIMs abolished CENP-B co-precipitation with Daxx (Fig. 2a). Next, we tested SIMs requirements for Daxx localization at centromeres in the same cellular models. As expected, in control conditions DaxxWT co-localized with centromeres (identified with CREST ab’s) in a subpopulation of cells and with ND10/PML bodies (identified with anti-PML antibodies) (Fig. 2b, upper row left). Association of DaxxWT with centromeres was elevated after proteasome block induced by treatment with MG132 (Fig. 2b, upper row right). However, DaxxΔSIM was not recruited to either nuclear location (centromeres or ND10/PML bodies) in control conditions or after proteasome block (Fig. 2b, bottom row). Thus, Daxx SIMs are required for association with CENP-B and, importantly, for Daxx targeting to centromeres, suggesting a SUMO-dependent recruitment mechanism.

### Both SUMO paralogs, SUMO-1 and SUMO-2/3, are associated with centromeres

DaxxΔSIM was not associated with CENP-B by IP and was not recruited to centromeres, while treatment with MG132, that increases sumoylation [38, 46], elevated accumulation of DaxxWT at centromeres (Figs. 1b, 2b). These data suggested a potential role of sumoylation in Daxx targeting to centromeres. In vertebrates, three major SUMO paralogs have been described: SUMO-1, SUMO-2 and SUMO-3. Human SUMO-2 and SUMO-3 are ∼ 96% identical and often referred to as SUMO-2/3, whereas they share only ~ 50% similarity with SUMO-1. SUMO-1 and SUMO-2/3 presumably serve distinct functions as they target different protein substrates and have distinctive intracellular dynamic and distribution [47]. To investigate the potential role of sumoylation in Daxx targeting to centromeres, we first probed for localization of SUMO paralogs at these domains. Immunofluoresce (IF) staining of MG132-treated HEp2 cells with SUMO-1 and SUMO-2/3 antibodies revealed the association of SUMO paralogs with centromeres (Fig. 3a).

### Expression of SENPs induces displacement of Daxx from centromeres

Sumoylation is a dynamic process regulated by SUMO-specific proteases (SENPs) that catalyze SUMO maturation/processing and SUMO de-conjugation from substrates [48]. Transient over-expression of either SENP1 or SENP2 abolished Daxx accumulation at centromeres (Fig. 3b). In addition, we observed differences between SENP1 and SENP2: expression of SENP1 (that does not have enzymatic preferences for de-conjugation toward SUMO paralogs 1 or 2/3 [48]) dispersed Daxx homogeneously within nucleoplasm, while expression of SENP2 (that has elevated de-conjugation activity to SUMO-2/3 modified substrates compared to SUMO-1 [48]) preserves Daxx association with non-centromeric domains, presumably at PML nuclear bodies, where PML is modified by both SUMO-1 or SUMO-2/3 [49, 50] for interaction with Daxx [34]. SENP-induced displacement of Daxx from centromeres further indicated that sumoylation of centromere substrate protein is required for Daxx targeting to centromeres. In addition, differences between SENP-1 and SENP-2 expression at Daxx intra-nuclear distribution suggests that both SUMO paralogs are involved in the localization of Daxx at PML nuclear bodies, while SUMO-2/3 modification mediates Daxx association with centromeres.

### SUMO-2 is required for Daxx association with CENP-B, for the recruitment of Daxx centromeres, and for association of H3.3 with centromeres

To test whether sumoylation is involved in the association between Daxx and CENP-B, we performed CENP-B IP from chromatin fraction of HEp2 cells depleted of either SUMO-1 or SUMO-2 (see Additional file 4: Fig. S4 top panel for efficiency of SUMO-1 and SUMO-2 depletion). Depletion of SUMO-1 did not affect association of Daxx with CENP-B, while depletion of SUMO-2 reduced it (Fig. 3c). SUMO-1 or SUMO-2 depletion does not affect levels of Daxx (Additional file 4: Fig. S4 bottom panel).

**Fig. 4:**
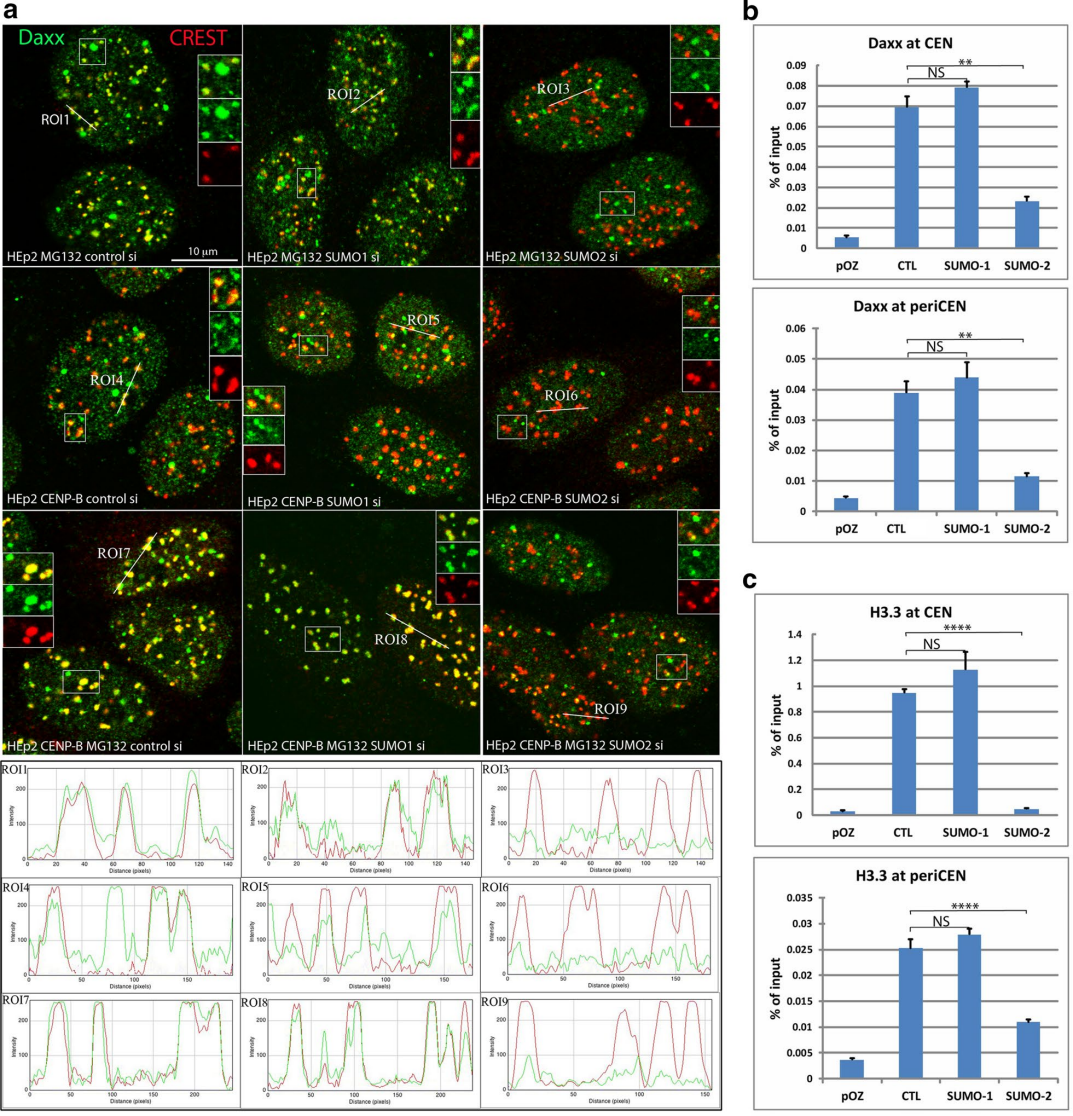
Targeting of Daxx and H3.3 at centromeres is SUMO-2 dependent. **a** SUMO-2 is required for Daxx association with centromeres. Representative images of HEp2 cells treated with MG132 (upper row) and FLAG-HA-CENP-B expressing HEp2 cells (untreated, middle row and MG132 treated, bottom row). SUMO-1 depletion (middle column) did not affect Daxx (green) accumulation at centromeres (CREST, red) compared to scramble siRNA transfection (left column), while SUMO-2 depletion (right column) abolished Daxx accumulation at centromeres in all three experimental conditions. Quantitation of the colocalization is presented in Additional file 3: Fig. S3. Graphs in the bottom panel show fluorescence intensity of Daxx (green) and CREST (red) signal along the ROI. **b** ChIP analysis of Daxx association with CEN and periCEN in HEp2 cells expressing FLAG-HA-Daxx. Depletion of SUMO-2, but not SUMO-1 abolished Daxx association with CEN and periCEN. **c** ChIP assay of H3.3 enrichment at CEN and periCEN in HEp2 cells stably expressing FLAG-HA-H3.3. Depletion of SUMO-2, but not SUMO-1, reduced H3.3 enrichment at CEN and periCEN. Bars represent the mean ± SD (*n* = 3). Statistical analysis was performed by one-way ANOVA followed by Tukey’s multiple comparisons test. ****P < 0.0001; ***P < 0.001; **P < 0. 01; *P < 0.1; NS, nonsignificant

IP results indicated contribution of SUMO-2 in CENP-B/Daxx association, suggesting a different role of SUMO paralogs in centromere recruitment of Daxx. To explore this possibility, we tested the effects of SUMO-1 and SUMO-2 depletion on Daxx accumulation at centromeres by microscopy analysis (Fig. 4a). Daxx accumulation at centromeres was not changed by transfection of neither scramble nor SUMO-1 siRNA in control HEp2 and in CENP-B expressing HEp2 cells, untreated or treated with proteasome inhibitor (Fig. 4a, left and middle columns, correspondingly), while depletion of SUMO-2 reduced Daxx association with centromeres in all tested conditions (Fig. 4a, right column and quantitation Additional file 3: Fig. S3). In addition, depletion of either SUMO-1 or SUMO-2 did not affect Daxx association with PML nuclear bodies (visible as bright domains of Daxx in all panels in Fig. 4a), further confirming that PML can be modified by either paralog for interaction with Daxx for recruitment to these domains.

To quantify SUMO dependence of Daxx recruitment to centromeres, we performed ChIP assay in chromatin fraction of HEp2 cells depleted of SUMO-1 or SUMO-2. Depletion of SUMO-2 reduced enrichment of Daxx at CEN (α-satellite repeats), confirming the necessity of this SUMO paralog for Daxx targeting to centromeres (Fig. 4b, top). Interestingly, similar effects were observed for Daxx recruitment at periCEN (HS3 pericentromeric repeats, Fig. 4b, bottom), suggesting a conserved role of SUMO-2 in Daxx localization at both centromere and pericentromere. In contrast, depletion of SUMO-1 did not reduce Daxx association with either CEN or periCEN repeats.

SUMO-2 mediates Daxx association with centromere and pericentromere repeats, suggesting contribution of SUMO-2 in H3.3 association with these genomic elements. Indeed, depletion of SUMO-2 reduced H3.3 association with both CEN and periCEN (Fig. 4c). Depletion of SUMO-1 did not reduce H3.3 levels at CEN and periCEN.

### CENP-B and Daxx deficiency increases chromosome instability

**Fig. 5:**
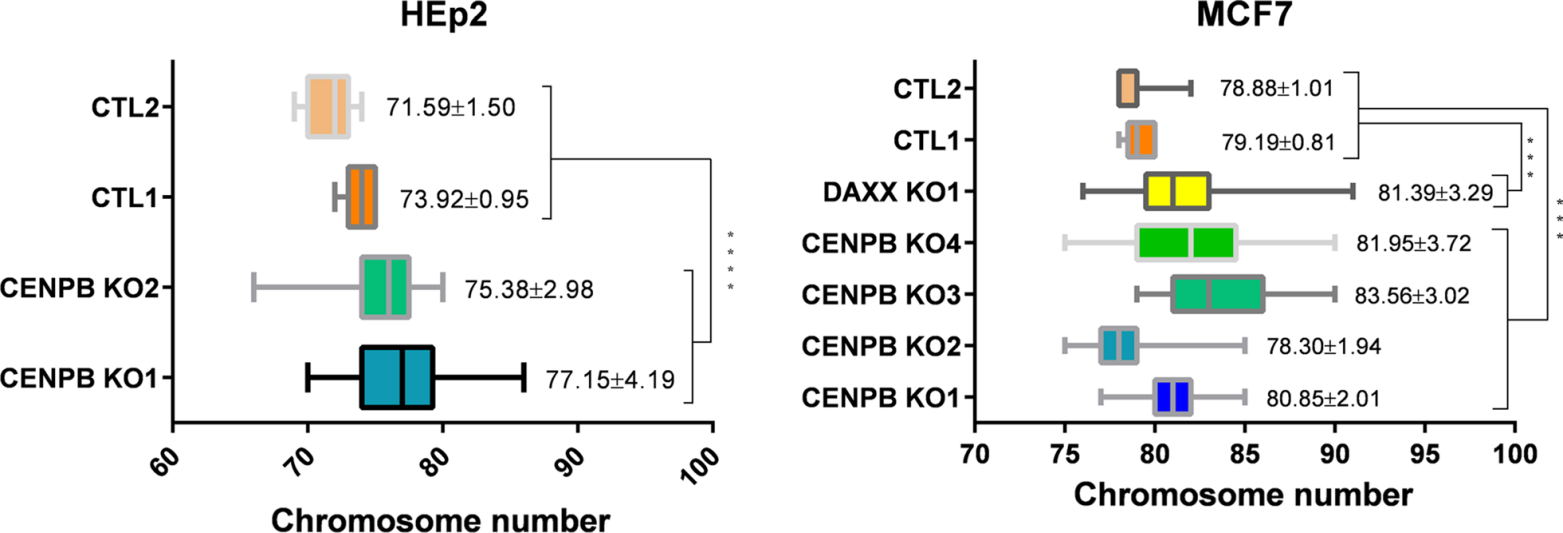
CENP-B and Daxx knockout increases chromosomal instability (CIN). Analysis of chromosome number in HEp2 and MCF7 cells; thick vertical line marks median of modal chromosome number, whiskers show *min* and *max*. Higher range of deviation from mean of modal chromosomal number was observed in CENP-B and Daxx knockout cell lines compared to control cells, that is indication of CIN. ***P < 0.001; ****P < 0.0001

So far, we demonstrated a novel CENP-B function in SUMO-2 and Daxx-mediated incorporation of histone H3.3 into centromere chromatin, suggesting a role for this constitutive centromere protein in chromatin maintenance. What are the physiological consequences of this incorporation? To answer this question, we employed CRISPR/Cas9 genome editing to produce CENP-B knockout (KO) HEp2 and MCF-7 cell lines (Additional file 5: Fig. S5A for cell lines characterization). While performing microscopy analysis of CENP-B and Daxx KO subclones, we noticed interphase nuclei abnormalities as micronuclei and nuclear blebs. These abnormalities are usually derived from mitotic segregation defects [51]. Indeed, we observed elevated levels of lagging chromosomes in MCF-7 CENP-B and Daxx KO clones (Additional file 5: Fig. S5B). These data suggest that CENP-B-depleted cells might have elevated chromosome instability (CIN). To address this possibility, we next analyzed the karyotypes of HEp2 and MCF-7 cells, in control and CENP-B KO clones. Karyotypes were nearly triploid for both analyzed parental cell lines, with modal chromosome number (calculated as an average number of chromosomes per mitotic plate for > 20 cells) of 72-74 for HEp2 cells (Fig. 5 left), and 79 for MCF-7 cells (Fig. 5 right). In all CENP-B KO clones, we observed two to almost four times increase of the standard deviation in chromosome number (Fig. 5). The changes in karyotypes resulted from chromosomes gain or loss are consistent with mitotic abnormalities and micronuclei formation observed in CENP-B KO cells, indicating elevated CIN in these cells. Similar karyotype changes were observed in Daxx KO MCF-7 cells (Fig. 5 right panel). Thus, knockout of CENP-B and its novel partner Daxx elevated CIN in human cells.

### CENP-B and Daxx deficiency deregulate heterochromatin at centromere

**Fig. 6:**
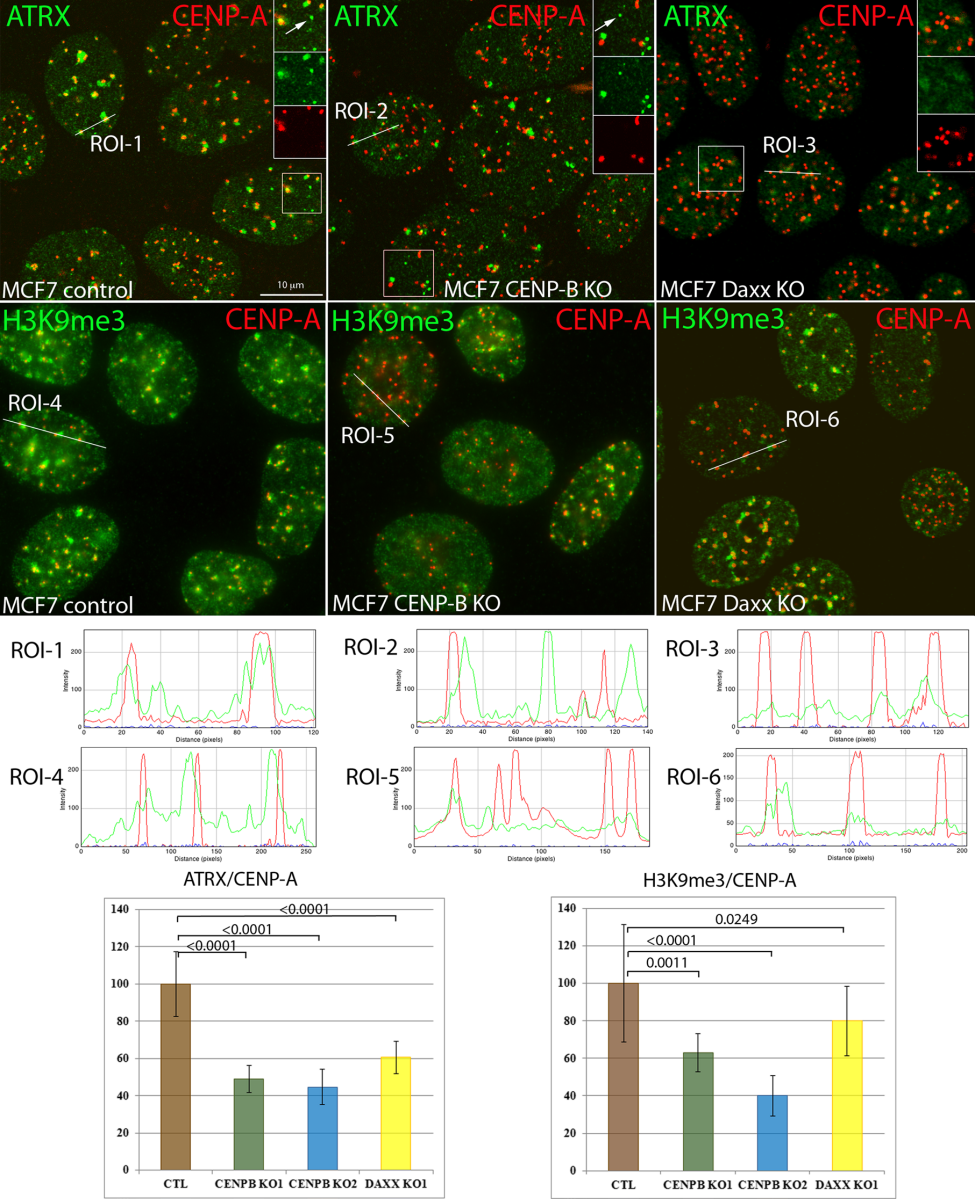
CENP-B and Daxx knockout reduce levels of ATRX and H3K9me3 at centromeres. Top: Representative images of MCF7 control (left column), CENP-B knockout (middle column), Daxx knockout (right column). Top row: ATRX (green) is associated with subpopulation of centromeres (CENP-A, red) and ND10/PML bodies (white arrow in inset) in MCF7 control cells (left); the association of ATRX with centromeres is reduced in CENP-B (middle) and Daxx (right) knockout cells. Note that there is no ND10-like ATRX staining in Daxx knockout cells since Daxx is required for ATRX recruitment to ND10/PML bodies [35]. Bottom row: CENP-B (middle) and Daxx (right) knockout reduce levels of H3K9me3 at centromeres when compared to control cells (left). Graphs in the bottom panel show fluorescence intensity along the region of interest (ROI). Bottom: Quantitative analysis of intensity of ATRX and H3K9me3 staining at centromeres. Data are shown relative to wild-type cells (100%). Statistical analysis was performed by one-way ANOVA followed by Tukey–Kramer multiple comparison test. The bars represent mean ± SD; > 200 cells from 10 random fields were analyzed for every clone

Epigenetics integrity of centromere chromatin is responsible for proper assembly and function of the kinetochore and attachments of spindle microtubules [52, 53]; when compromised, it results in CIN, as we observed in CENP-B KO cells. This CIN may derive from several abnormalities previously observed after CENP-B depletion, including mislocalization of CENP-A and CENP-C [25, 28] and problems with centromere DNA replication [27]. Yet, we also observed similar levels of CIN in Daxx KO cells, suggesting functionality of CENP-B/Daxx association in the maintenance of genomic stability. In addition, previous work has shown that H3.3 double KO compromised chromatin structure at telomeres, centromeres and pericentromeres leading to increased mitotic abnormalities such as anaphase bridges and lagging chromosomes in ES and MEF cells [54]. Thus, deficiency of H3.3 at centromere induces CIN phenotypes similarly to what we observed in CENP-B and Daxx KO cells, further pointing at potential function of CENP-B/Daxx-dependent H3.3 pathway in the epigenetics integrity of centromere chromatin. To address this hypothesis, we first analyzed centromere association of Daxx interaction partner and member of Daxx-dependent H3.3 chaperone complex protein ATRX. alpha thalassemia/mental retardation syndrome X-Linked (ATRX) is the SWI/SNF chromatin remodeling ATP-dependent helicase; loss or mutations of ATRX resulted in CIN, through abnormal centromeric mitotic recombination and ds-DNA breaks [55–58]. Daxx interacts with ATRX and recruits ATRX in ND10/PML nuclear bodies [35], while, at least in mouse fibroblasts, ATRX recruits Daxx to the pericentromeric heterochromatin (comprised of major satellite repeats in mouse) at the end of S-phase [35]. Thus, we tested ATRX localization in CENP-B KO and Daxx KO MCF-7 cells. In control cells, ATRX co-localized with centromeres (identified with CENP-A antibodies, Fig. 6, left top row, and ROI-1) and accumulated in additional domains, most likely representing the PML nuclear bodies (Fig. 6 left top row, insert, white arrow). We observed reduction of ATRX accumulation at centromeres in CENP-B KO cells (Fig. 6, middle top and ROI-2); as expected, CENP-B deletion did not affect ATRX accumulation at PML nuclear bodies (Fig. 6 middle top row, insert, white arrow). Knockout of Daxx also reduced ATRX association with centromeres (Fig. 6, right top row, ROI-3) and abolished ATRX accumulation at PML nuclear bodies patterns as observed previously [35]. Quantification analysis revealed 45% and 51% reduction of ATRX association with centromeres in two CENP-B-deleted clones and 40% reduction in Daxx-deleted cells compared to control cells (Fig. 6 bottom panel, left graph). Next, we tested heterochromatin-associated epigenetic marker H3K9me3, which is generally enriched at centromeres and pericentromeres [59]. Levels of H3K9me3 at centromeres were drastically reduced in CENP-B-deleted cells (Fig. 6 middle bottom row and right graph). We also found reduction of H3K9me3 at centromeres in Daxx-deleted cells (Fig. 6 right bottom row and right graph); difference in H3K9me3 reduction between CENP-B and Daxx KO suggests that CENP-B may have an additional effect on H3K9me3, for instance, via recruitment of SUV39h. Additionally, knockout of CENP-B reduced accumulation of the key heterochromatin protein HP1α at centromeres (Additional file 6: Fig. S6 right top row and ROI-2) and reduced the co-localization of HP1α and ATRX at these structures (Additional file 6: Fig. S6 right middle row and ROI-4). Thus, deletion of constitutive centromere protein CENP-B and its novel partner, H3.3 chaperone protein Daxx, deregulates centromeric heterochromatin, reducing association of two heterochromatin proteins, ATRX and HP1α, and also diminishing heterochromatin epigenetic marker, H3K9me3 at centromeres. Together, our data indicate that CENP-B/Daxx-dependent H3.3-loading pathway contributes in the protection of epigenetic landscape at centromeres for preservation of genomic stability, while deregulation of this pathway induces CIN.

## Discussion

**Fig. 7:**
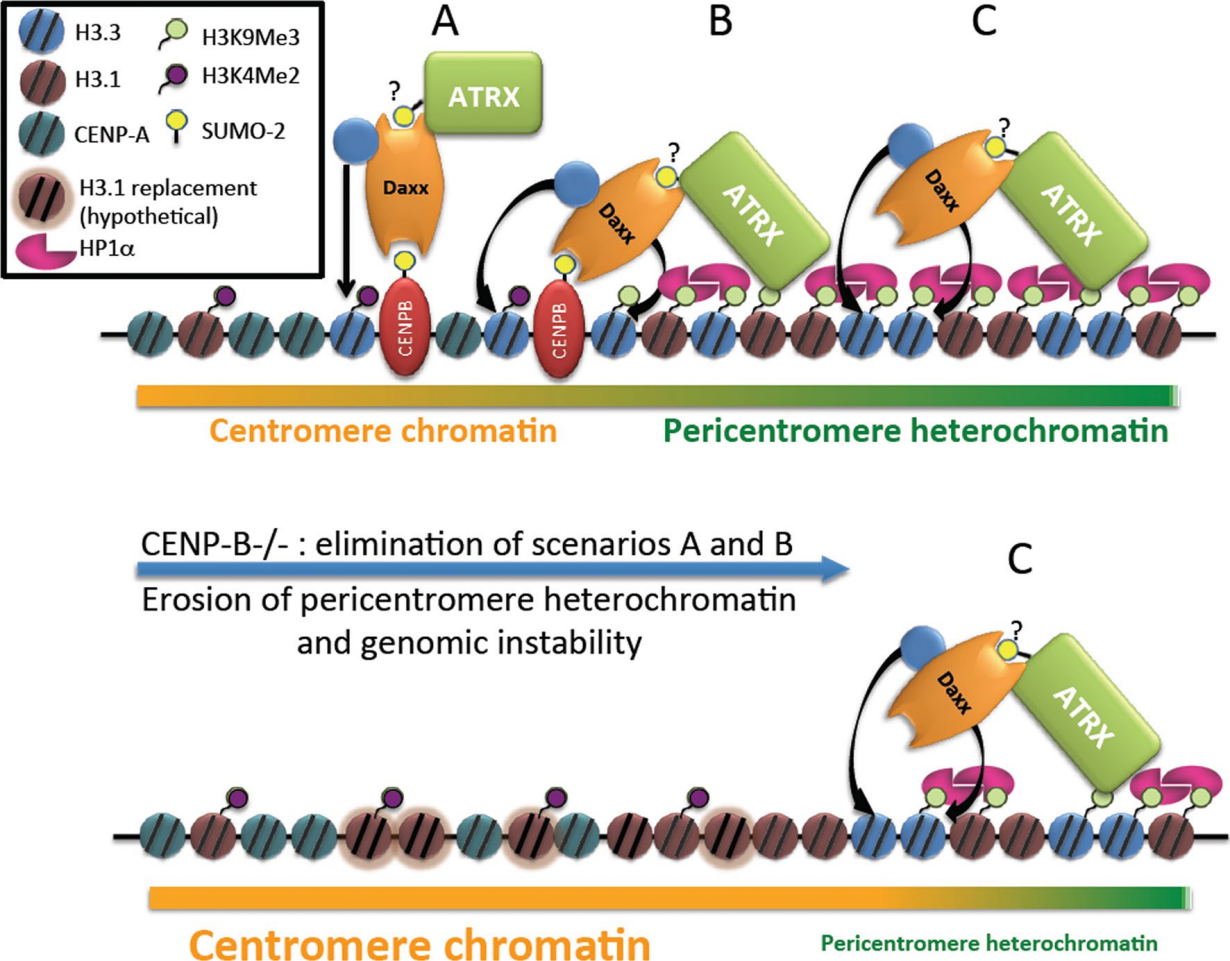
Model of CENP-B/Daxx-mediated loading of H3.3 for centromere/pericentromere chromatin balance. A schematic linear representation of a centromeric and pericentromeric chromatin. Zone “A”: centromere; Daxx complex is recruited by interaction of Daxx SIM with SUMO-2-modified CENP-B; Daxx-dependent H3.3 loading promotes H3K9 trimethylation in vicinity of CENP-B, potentially through SUV39h1-dependent mechanism. Zone “C”: pericentromere; Daxx complex is recruited possibly by interaction of ATRX with H3K9Me3 that is enriched at pericentromere. Zone: “B”: transient/border area. Disruption of CENP-B/Daxx-dependent loading of H3.3 to centromere by knocking out CENP-B or Daxx leads to heterochromatin erosion that proceeds from proximal toward distal region of pericentromere. Sumoylation-dependent interaction between Daxx and ATRX is speculative and indicated by question mark

In this study, we identified the constitutive centromere protein CENP-B as a new interaction partner of H3.3 chaperone Daxx. This interaction requires for H3.3 loading at centromeres, thus assigning a novel function for CENP-B. Our results also imply that SUMO-2 mediates accumulation of Daxx and association of H3.3 at both centromeres and pericentromeres. Summarizing these observations, we propose a three-zone model for H3.3 loading and the maintenance of the centromere epigenetic landscape (Fig. 7). In zone “A,” CENP-B is anchored at α-satellite HORs and α-satellite dimers [18]. Interaction between CENP-B (most likely modified by SUMO-2) and Daxx SIMs recruits Daxx/ATRX complex for H3.3 loading. In zone “C,” Daxx/ATRX complex is recruited potentially by ATRX interaction with H3K9me3 for H3.3 loading. This model is indeed over-simplified, as it does not include all the players of centromere maintenance. In addition, it excludes 3D aspect of centromere/pericentromere chromatin organization such as the amphipathic helixes/loops model or the recently proposed boustrophedon model [12], which postulates close 3D proximity and reciprocal influence of zones “A” and “C.” Yet, even in the linear form, our model suggests existence of transition zone “B” that can be affected by changes in zones “A” or “C” and can be stochastically shifted in both directions as a result of these changes. We were able to register a final result of these sequential changes as an erosion of heterochromatin (i.e., reduction of H3K9me3, ATRX and HP1α) in CENP-B and Daxx knockout cells (Fig. 6 and Additional file 6: Fig. S6). A recent publication identified similar function of Daxx in mouse cells: Daxx knockout [35] induced structural alteration of chromatin and separation of H3K9me3 from the major satellite DNA [60]. Importantly, structure of the pericentromeric domain is necessary for the cohesion-mediated chromatid connection that provides elasticity and resistance to tension during mitosis [61–63]. Thus, disorganization of pericentromeres can induce chromosomal instability (CIN), as we observed here (Fig. 5).

Recent study of H3.3-null mouse reveals an essential function of H3.3 in the protection of heterochromatin structure and stability. Knockout of both H3.3 genes triggers dysfunction of heterochromatin at telomeres, centromeres and pericentromeric regions, leading to mitotic abnormalities and cell death [54]. Thus, CIN observed in CENP-B knockout human cells ([28] and this study Fig. 5) can be, at least partly, attributed to dis-regulation of H3.3 pathway at centromeres; as a confirmation, we identified similar CIN in cells that are deleted for H3.3 chaperon Daxx (Fig. 5). Notably, previous observations that CENP-B null mice had a minor phenotype [64–66], leading to the ambiguous conclusions regarding functionality of CENP-B that were recently challenged by the observation that mouse CENP-B knockout cells have a high rate of CIN [28]. Mice generally tolerate somatic aneuploidy well (discussed in [28]), that may, indeed, result in minor phenotype of CENP-B null mouse models. In addition, recent work demonstrated that CENP-A regulates CENP-B stabilization at centromeres [25] that, in combination with our results of CENP-B-dependent H3.3 loading, can provide a potential feedback mechanism for the maintenance of centromere chromatin. H3.3-enriched chromatin is associated with elevated transcription, including noncoding transcripts expressed from the α-satellite repeats [7, 32]. These noncoding RNA are involved in the heterochromatin formation [67], suggesting transcription-associated role of H3.3 in the maintenance of centromeric chromatin. H3.3 can also serve as a CENP-A placeholder after DNA replication as it forms more “loose” nucleosomes for the subsequent replacement in M/G1 by CENP-A containing nucleosomes [68]. Thus, H3.3 may participate in centromere epigenetics via several mechanisms, including noncoding transcription and proper CENP-A incorporation. Pericentromere is assembled by multiple events, including SUV39h-mediated H3K9 methylation, that recruits heterochromatic proteins HP1α, that, in addition, can be recruited by interaction with satellite-transcribed RNA [69–71]. H3K9me3 interacts and recruits ATRX/Daxx for H3.3 loading that elevates satellite-transcribing RNA, thus, potentially, boosting HP1α recruitment.

Our results suggest that CENP-B maintains epigenetic landscape of centromere/pericentromere by coupling CENP-B box positive α-satellite repeats, Daxx-mediated loading of histone H3.3, and H3K9me3 modification, potentially through attraction of SUV39h [23, 72]. Thus, CENP-B associated with α-satellite repeats can provide center of nucleation for H3K9me3 modification from which this mark can spread further toward the pericentromeric region through the positive feedback mechanism, attracting ATRX together with SUV39h/SETDB1 as it has been recently shown for telomeres [72] and retroviral elements [73].

Chromatin organization elements at centromere can be categorized according to “fixed” and “negotiable” borders hypothesis [74]. According to this model, CENP-B, associated with CENP-B boxes at α-satellite repeats, may serve as a keystone for establishing “fixed” centromere/pericentromere epigenetic border that prevents spread of centromere histone modification to pericentromere heterochromatin and vice versa. Our model is in agreement with one proposed by Okada et al. [23], who suggested CENP-B-dependent dynamic balance between CENP-A and H3K9me3. According to this model, CENP-B may perform antagonistic roles by assembling CENP-A at centromere region and by elevating heterochromatic H3K9me3 modification at pericentromere region, and, thus, can be viewed as a border protein separating these regions of chromatin. Importantly, CENP-B is associated with the kinetochore and also with the inner centromere [29–31], further confirming its bordering position and dualistic functions of CENP-B at these locations.

CENP-B knockout eliminates “fixed” border, but epigenetics can be maintained, to some extent, via hypothetical “negotiable” mechanism although it is not as effective since exact position of the border is not determined. Naturally occurring examples of this scenario are Y chromosome and neocentromeres that lack B-boxes and therefore CENP-B. In both cases, CENP-B negative chromosomes mis-segregate with higher frequency than normal centromeres with CENP-B boxes [28], that may be explained by the absence of “fixed” border mechanism and dysfunction of kinetochores. In this study, we observed similar effect in CENP-B knockout cells. Interestingly, the downstream effect of CENP-B/Daxx-mediated H3.3 pathway disruption (as CIN and heterochromatin erosions) was observed only in knockout cells that undergo multiple cell divisions. In addition, we observed heterogeneity in heterochromatin markers erosions within cell population (Fig. 6 and Additional file 6: Fig. S6). These data may be interpreted as evidence of the stochastic loss of chromatin equilibrium in the absence of “fixed” border.

A surprising finding is that SUMO-2, but not SUMO-1, promotes accumulation of Daxx at centromeres. While a common enzymatic machinery is involved in conjugation of all SUMO paralogs [75], accumulating body of evidence points to functional differences between SUMO-1 and SUMO-2/3 modifications and pathways [76]. Although many substrates can be covalently modified by both SUMO-1 and SUMO-2/3, SUMO-1 is mostly found in the conjugated form, while SUMO-2/3 are primarily found free and thus can be rapidly conjugated to substrates in response to a number of stress stimuli [77]. Consequently, SUMO-2 conjugation is more dynamic than SUMO-1 [78] and changes throughout the cell cycle [79]. Here, we provide the first evidence that SUMO-2 participates in the chromatin landscape maintenance by targeting H3.3 to centromeres. Several studies identified CENP-B as a SUMO-2 substrate in proteomic screens [80, 81]. These studies also observed increased CENP-B modification by SUMO-2 after proteasome inhibition or heat shock, that can explain elevated accumulation of Daxx at centromeres after treatment with the proteasome inhibitor MG132 (Fig. 1 and [36]), or application of hyperthermia [32]. Finally, another recent proteomic screen uncovered dynamic changes in CENP-B sumoylation during cell cycle progression with maximum in S-phase [79]. It is tempting to speculate that CENP-B can be modified by SUMO-2 during DNA replication; this, in turn, can attract Daxx at centromeres during S-phase (as we previously demonstrated in mouse cells [35]) to facilitate its H3.3 chaperon activity by gap-filling mechanism [82].

Our data suggested that Daxx-containing H3.3 chaperone complex is targeted to the zone “A” and zone “C” differently; by CENP-B to “A,” and by another partner to “C,” but in both cases this targeting is mediated by SUMO-2. Further studies are required to identify protein(s) responsible of Daxx recruitment to “C.” A potential candidate is a component of H3.3 chaperone complex, protein ATRX. This protein interacts with Daxx [35, 83], is modified by SUMO-2 [80] and can be targeted to pericentromere by interaction with (1) heterochromatin protein HP1α [84], (2) H3K9me3, enriched at pericentromere chromatin [85], and (3) pericentromeric G-quadruplexes DNA structures [86].

What mechanism determines Daxx specificity toward SUMO-2-modified target (presumably CENP-B) at centromere? Two groups reported that Daxx binds SUMO-1 and SUMO-2 with similar affinity [45, 87], though phosphorylation of Daxx SIM by CK2 enhances affinity toward SUMO-1 over SUMO-2 [88]. Thus, it is plausible to propose that yet an unidentified posttranslational modification of Daxx (potentially, cell cycle dependent) may determine higher affinity to SUMO-2 modified partners and/or can affect the interaction between Daxx and H3.3. Potentially, distinctive Daxx modifications can modulate Daxx SIMs affinity toward one or another SUMO paralog and therefore regulate targeting of Daxx to different nuclear compartments; this assumption is awaiting further investigation.

## Conclusions

CENP-B is the only centromere protein that binds specific DNA sequences (CENP-B boxes) at centromere repeats, yet the function of this protein remains elusive. Here, we demonstrate that CENP-B serves as a “beacon” for SUMO-dependent recruitment of Daxx chaperone complex that incorporates histone variant H3.3 into CENP-B box-containing centromere repeats. Functionally, disruption of CENP-B/Daxx-dependent H3.3-loading pathway deregulates epigenetic integrity of centromeres and elevates chromosome instability. These studies highlight CENP-B as a potential bond between two centromere elements, DNA and chromatin, that establishes histone variant landscape at centromere for protection of genome stability.

## Methods

### Cell culture

HEp2 (human epithelial type 2, laryngeal carcinoma) and MCF7 (breast cancer) cells were cultured in Dulbecco’s modified Eagle’s medium (DMEM) supplemented with 10% fetal bovine serum, 2 mM glutamine and 100 U/mL penicillin and 100 µg/mL streptomycin (Gibco BRL, Carlsbad, CA) and grown at 37 °C in a humidified 5% CO2 incubator. HEp2 cells expressing FLAG-HA-Daxx, DaxxΔSIM and CENP-B were created using pOZ expression vector [89] as described in [32]. Proteasome inhibitor MG132 (Cayman Chemical, Ann Arbor, Michigan) was dissolved in DMSO (Sigma, St. Louis, MO) and used at final concentration 10 µM for 4 h.

### Antibodies

Daxx 677B rabbit and Daxx 5.14 monoclonal [35], PML-14 rabbit [34], ATRX rabbit (#sc-15408, Santa Cruz Biotechnology, Santa Cruz, CA), CENP-B rabbit (#sc-22788, Santa Cruz), CENP-B rabbit (#ab25734, Abcam), CENP-B mouse monoclonal (gift from Dr. Masumoto, Kazusa DNA Research Institute), CENP-C mouse monoclonal (#ab50974, Abcam), actin mouse monoclonal (Sigma), HA mouse monoclonal (Covance, Emeryville, CA), SUMO-1 rabbit were obtained from Dr. P. Freemont (ICRF London) [90], SUMO-2 rabbit (#51-9100, Zymed), CREST human autoimmune antibodies [34].

### Immunoprecipitation

Cells were washed twice with cold PBS buffer supplemented with 5 mM iodoacetamide (Sigma) and 1 mM phenylmethylsulfonylfluoride (Sigma). Cells were scraped directly in 1 ml lysis buffer (50 mM Tris–HCL (pH 7.4), 150 mM NaCl, 1 mM EDTA, 1% Triton X-100) in the presence of 10 mM N-ethylmaleimide (Sigma), 5 mM iodoacetamide (Sigma), 1 mM phenylmethylsulfonylfluoride (Sigma), 1 mg/mL aprotinin (Sigma), 1 mM leupeptin (Sigma), 1 mM pepstatin (Sigma), transferred into Eppendorf tube and incubated 10 min, 4 °C, with rotation. Lysate was centrifuged at 1200*g* for 10 min, 4C. Pellet was resuspended in lysis buffer supplemented with 400 mM NaCl (high salt buffer, HSB); chromatin fraction was extracted for 30 min with rotation at 4 °C. The pellet was briefly sonicated using Misonix Sonicator 3000 (2 cycles 10 s on/50 s off, power 2.5) in the experiments with MG132 treatment. Extract was pre-cleared by centrifugation at 16,000*g* for 10 min, 4 °C and incubated with preconditioned FLAG magnetic beads (Sigma) for 4 h, 4 °C with rotation. Beads were washed six times with HSB and eluted with FLAG peptide.

### Western blotting

Protein samples were separated by 4–20% SDS-PAGE (Bio-Rad), transferred to nitrocellulose membrane (Whatman, Dassel, Germany) and blocked with 5% nonfat milk/PBS, 0.1% Tween (PBST). Primary antibodies were diluted in 5% milk/PBST and incubated overnight at 4 °C. Membrane was washed 3 times with PBST and incubated for 1 h at RT with appropriate secondary antibody (Vector Laboratories, Burlingame, CA). Membrane was washed 3 times with PBST and visualized by enhanced chemiluminescence (ECL). Densitometry analysis of Western blots was performed using the ImageJ software (Rasband, WS, ImageJ, U. S. National Institutes of Health, Bethesda, Maryland, USA, http://imagej.nih.gov/ij/, 1997–2014.)

### Chromatin immunoprecipitation (ChIP)

ChIP was performed as described in [32]. Briefly, formaldehyde was added directly to cell media to final concentration 1%. The reaction was quenched after 10 min by adding glycine to final concentration 0.125 M for 5 min. Cells were washed twice with PBS, resuspended in lysis buffer and incubated at 4 °C for 10 min with rotation. Cells were centrifuged at 1200*g* for 5 min; supernatant was discarded and pellet was resuspended in HSB IP buffer, transferred into 5 ml Falcon round-bottom tube and sonicated on ice using Misonix Sonicator 3000 (6 cycles 10 s on/50 s off, power 2.5) with 0.5-mm glass beads (BioSpec Products). Supernatant was transferred in Eppendorf tube, and insoluble material was removed by centrifugation at 16,000*g* for 10 min, 4 °C. Chromatin was immunoprecipitated with anti-FLAG M2 magnetic beads (Sigma) for 2 h with rotation. Beads were washed 5 times with HSB and finally with TE buffer. Chromatin was eluted with a buffer containing 10 mM Tris pH 8.0, 1% SDS, 5 mM EDTA at 65 °C for 30 min. Cross-link was reversed by the addition of 5 M NaCl and incubation at 65 °C for 4 h. The samples were diluted with equal volume of TE buffer and treated with proteinase K for 2 h. DNA was purified by phenol/chloroform extraction and ethanol precipitation and analyzed by quantitative PCR for α-satellite centromere repeats (CEN) and satellite III repeats chromosome 9 (periCEN) using StepOne RT-PCR machine (Applied Biosystems) as described in [32].

### Immunofluorescence

Cells were grown on microscope cover glass (Fisher Scientific, Pittsburgh, PA) in 24-well plate (Corning Inc., Lowell, MA USA). Cells were fixed with 1% formaldehyde (Fisher Scientific) in PBS for 10 min, permeabilized with 0.5% Triton/PBS for 14 min on ice and then incubated for an hour at RT with primary antibodies followed by wash with PBS and visualization with highly cross-absorbed secondary antibodies conjugated with Alexa Fluor 488, 594 or 647 dye (Invitrogen, Carlsbad, CA). Cells were stained with Hoechst 33342 (Sigma) for DNA visualization and mounted on slides (Fisher Scientific) with Fluoromount G (Southern Biotech, Birmingham, AL, USA). Images were analyzed using Leica TCS SP5 confocal microscope.

Quantitative analysis of IF images was performed using Volocity 6.3 software (PerkinElmer). At least 200 cells from 10 randomly picked up fields were analyzed for every clone. Statistical analysis was done by GraphPad Prism software (GraphPad Software, Inc., La Jolla, CA).

### Metaphase chromosomes spread

Cells were treated with 100 ng/ml colcemid (Invitrogen) for 4 h before proceeding with metaphase preparations. Cells harvested by shack-off were resuspended in hypotonic buffer (10 mM Tris–HCl pH 7.4, 40 mM glycerol, 20 mM NaCl, 5 mM KCl, 1 mM CaCl, 0.5 mM MgCl_2_) 2 × 10^4^ cells/300 µl and incubated for 15 min on ice. 2 × 10^4^ cells were cytocentrifuged, fixed with ethanol/acetone (1:1) or 1% formaldehyde.

### siRNA transfection

2 × 10^5^ HEp2 cells were transfected with two rounds 100 nM siRNA (GE Healthcare Dharmacon, Lafayette, CO) using DharmaFect 1 transfection reagent (GE Healthcare Dharmacon). Cells were used for experiments 48 h after 2nd round of transfection. The following siRNA were used: SUMO-1 D-016005-05, smart pool SUMO-2 L-016450-00-0005, smart pool Daxx L-004420-00-0050, smart pool ATRX D-006524-01-0005, smart pool CENP-B L-003250-00-0005 and smart pool CENP-C L003251-00-0005.

### CENP-B and Daxx knockout by CRISPR/Cas9 system

Two pairs of oligonucleotides for each gene were design to produce deletion in Daxx and CENP-B: (5′-*CACCG* **TGGTCCCATTCCTCTATAAC** and 5′-*CACCG* **GATGTTGCAGAACTCCGCCG**) for Daxx and for (5′-*CACCG* **TGCGCAAGGGCGAGATCGCG** and 5′-*CACCG* **CGTACTTGCGCTCCGACGCC**) for CENP-B. Oligos were ligated into BsmBI site of lentiCRISPR vector [91] (Addgene, 49535). Cells were transfected with the sgRNA expression vectors using Lipofectamine 2000 (Invitrogen, Carlsbad, CA), on the third day after transfection growth media was supplemented with 2 µg/ml (HEp2) or 0.5 µg/ml (MCF7) puromycin. After five days of selection, survived cells were transferred in 96-well plates for clonal growth. Clones were expended and analyzed by PCR, western blot and immunofluorescence.

## Additional files

**Additional file 1: Fig. S1.** Association of CENP-B and Daxx in chromatin fraction (relevant to Fig. 1a). **A**. FLAG IP from chromatin fraction of HEp2 cells expressing FLAG-HA-Daxx probed with CENP-B antibodies. **B**. Reciprocal IP from chromatin fraction of HEp2 cells expressing FLAG-HA-CENP-B probed with Daxx antibodies.

**Additional file 2: Fig. S2.** CENP-B and CENP-C depletion efficiency (relevant to Fig. 1b). Representative images of HEp2 cells transfected with scrambled (control), CENP-B and -C siRNA and stained for CENP-C (green) and CREST (red) (top and second rows) or CENP-B (green) and CENP-A (red) (third and bottom rows); DNA blue. Both siRNAs significantly reduced levels of corresponding centromere proteins.

**Additional file 3: Fig. S3.** Quantitative analysis of Daxx intensity staining at centromeres (relevant to Figs. 1b and 4a). Data in arbitrary units for control-, CENP-B-, CENP-C-, SUMO-1-, and SUMO-2-depleted cells after MG132 treatment of HEp2 cells. Statistical analysis was performed by one-way ANOVA followed by Dunnett’s multiple comparisons test. The bars represent mean ± SD. P < 0.001: ***; P < 0. 01: **; NS: nonsignificant.

**Additional file 4: Fig. S4.** Top: SUMO depletion efficiency (relevant to Fig. 4). HEp2 cells expressing FLAG-HA-CENP-B (pOZ-CENP-B) were transfected with scrambled siRNA (CTL), SUMO-1 or -2 siRNA and treated with MG132 for 4 h. Cell lysates were analyzed by Western blot and probed with SUMO-1 (left) or SUMO-2 (right) antibodies. Control: un-transfected HEp2 cells (pOZ). Both SUMO siRNA reduced levels of corresponding conjugates. Bottom: SUMO depletion does not change levels of endogenous Daxx (relevant to Fig. 3c). Conditions as above; chromatin-associated fractions were analyzed by Western blot and probed with Daxx antibodies. *: unspecific band. Control: un-transfected HEp2 cells (pOZ). SUMO-1 or -2 depletion does not affect levels of Daxx.

**Additional file 5: Fig. S5.** **A**. Western blot analysis of HEp2 and MCF7 CENP-B and Daxx knockout clones. **B**. Analysis of micronuclei and lagging chromosomes in MCF-7 knockout clones. At least 100 mitotic events were analyzed for each clone.

**Additional file 6: Fig. S6.** CENP-B and Daxx knockout reduce HP1α accumulation at centromeres. Representative images of MCF7 control (left column) and CENP-B knockout (right column). Top row and ROI-1/-2: knockout of CENP-B (right) reduced accumulation of key heterochromatin protein HP1α (green) at centromeres (CREST, blue). Bottom row and ROI-4: knockout of CENP-B (right) reduced co-localization of HP1α (green) and ATRX (red) at centromeres (CREST, blue). PML nuclear bodies marked with arrows.

## Abbreviations

CENP-B: centromere protein B
ChIP: chromatin immunoprecipitation
HORs: higher-order repeat units
ND10: nuclear domain 10
ROI: region of interest
SIM(s): SUMO interaction motifs(s)
SUMO: small ubiquitin-like modifier
TR: tandem DNA repeats

## References

[1] Buschbeck M, Hake SB (2017). Variants of core histones and their roles in cell fate decisions, development and cancer. Nat Rev Mol Cell Biol.

[2] Hake SB, Allis CD (2006). Histone H3 variants and their potential role in indexing mammalian genomes: the “H3 barcode hypothesis”. Proc Natl Acad Sci USA.

[3] Szenker E, Ray-Gallet D, Almouzni G (2011). The double face of the histone variant H3.3. Cell Res.

[4] Talbert PB, Henikoff S (2017). Histone variants on the move: substrates for chromatin dynamics. Nat Rev Mol Cell Biol.

[5] Ahmad K, Henikoff S (2002). Histone H3 variants specify modes of chromatin assembly. Proc Natl Acad Sci USA.

[6] Lewis PW, Elsaesser SJ, Noh KM, Stadler SC, Allis CD (2010). Daxx is an H3.3-specific histone chaperone and cooperates with ATRX in replication-independent chromatin assembly at telomeres. Proc Natl Acad Sci USA.

[7] Drane P, Ouararhni K, Depaux A, Shuaib M, Hamiche A (2010). The death-associated protein DAXX is a novel histone chaperone involved in the replication-independent deposition of H3.3. Genes Dev.

[8] Ivanauskiene K (2014). The PML-associated protein DEK regulates the balance of H3.3 loading on chromatin and is important for telomere integrity. Genome Res.

[9] Salomoni P (2013). The PML-interacting protein DAXX: histone loading gets into the picture. Front Oncol.

[10] Pesenti ME, Weir JR, Musacchio A (2016). Progress in the structural and functional characterization of kinetochores. Curr Opin Struct Biol.

[11] Nagpal H, Fukagawa T (2016). Kinetochore assembly and function through the cell cycle. Chromosoma.

[12] Fukagawa T, Earnshaw WC (2014). The centromere: chromatin foundation for the kinetochore machinery. Dev Cell.

[13] Marshall OJ, Chueh AC, Wong LH, Choo KH (2008). Neocentromeres: new insights into centromere structure, disease development, and karyotype evolution. Am J Hum Genet.

[14] Earnshaw WC, Rothfield N (1985). Identification of a family of human centromere proteins using autoimmune sera from patients with scleroderma. Chromosoma.

[15] Sullivan BA, Karpen GH (2004). Centromeric chromatin exhibits a histone modification pattern that is distinct from both euchromatin and heterochromatin. Nat Struct Mol Biol.

[16] Lam AL, Boivin CD, Bonney CF, Rudd MK, Sullivan BA (2006). Human centromeric chromatin is a dynamic chromosomal domain that can spread over noncentromeric DNA. Proc Natl Acad Sci USA.

[17] Alexandrov I, Kazakov A, Tumeneva I, Shepelev V, Yurov Y (2001). Alpha-satellite DNA of primates: old and new families. Chromosoma.

[18] Henikoff JG, Thakur J, Kasinathan S, Henikoff S (2015). A unique chromatin complex occupies young alpha-satellite arrays of human centromeres. Sci Adv.

[19] Van Hooser AA, Mancini MA, Allis CD, Sullivan KF, Brinkley BR (1999). The mammalian centromere: structural domains and the attenuation of chromatin modeling. FASEB J.

[20] Blower MD, Sullivan BA, Karpen GH (2002). Conserved organization of centromeric chromatin in flies and humans. Dev Cell.

[21] Masumoto H, Masukata H, Muro Y, Nozaki N, Okazaki T (1989). A human centromere antigen (CENP-B) interacts with a short specific sequence in alphoid DNA, a human centromeric satellite. J Cell Biol.

[22] Tanaka Y (2005). Human centromere protein B induces translational positioning of nucleosomes on alpha-satellite sequences. J Biol Chem.

[23] Okada T (2007). CENP-B controls centromere formation depending on the chromatin context. Cell.

[24] Suzuki N (2004). CENP-B interacts with CENP-C domains containing Mif2 regions responsible for centromere localization. J Biol Chem.

[25] Fachinetti D (2013). A two-step mechanism for epigenetic specification of centromere identity and function. Nat Cell Biol.

[26] Nakano M (2008). Inactivation of a human kinetochore by specific targeting of chromatin modifiers. Dev Cell.

[27] Erliandri I (2014). Replication of alpha-satellite DNA arrays in endogenous human centromeric regions and in human artificial chromosome. Nucleic Acids Res.

[28] Fachinetti D (2015). DNA sequence-specific binding of CENP-B enhances the fidelity of human centromere function. Dev Cell.

[29] Warburton PE (1997). Immunolocalization of CENP-A suggests a distinct nucleosome structure at the inner kinetochore plate of active centromeres. Curr Biol.

[30] Ohzeki J, Nakano M, Okada T, Masumoto H (2002). CENP-B box is required for de novo centromere chromatin assembly on human alphoid DNA. J Cell Biol.

[31] Politi V (2002). CENP-C binds the alpha-satellite DNA in vivo at specific centromere domains. J Cell Sci.

[32] Morozov VM, Gavrilova EV, Ogryzko VV, Ishov AM (2012). Dualistic function of Daxx at centromeric and pericentromeric heterochromatin in normal and stress conditions. Nucleus.

[33] Jarzabek-Chorzelska M (1990). Antikinetochore and antitopoisomerase I antibodies in systemic scleroderma: comparative study using immunoblotted recombinant antigens, immunofluorescence, and double immunodiffusion. Arch Dermatol Res.

[34] Ishov AM (1999). PML is critical for ND10 formation and recruits the PML-interacting protein daxx to this nuclear structure when modified by SUMO-1. J Cell Biol.

[35] Ishov AM, Vladimirova OV, Maul GG (2004). Heterochromatin and ND10 are cell-cycle regulated and phosphorylation-dependent alternate nuclear sites of the transcription repressor Daxx and SWI/SNF protein ATRX. J Cell Sci.

[36] Everett RD (1999). A dynamic connection between centromeres and ND10 proteins. J Cell Sci.

[37] Pluta AF, Earnshaw WC, Goldberg IG (1998). Interphase-specific association of intrinsic centromere protein CENP-C with HDaxx, a death domain-binding protein implicated in Fas-mediated cell death. J Cell Sci.

[38] Matafora V, D’Amato A, Mori S, Blasi F, Bachi A (2009). Proteomics analysis of nucleolar SUMO-1 target proteins upon proteasome inhibition. Mol Cell Proteomics.

[39] Muro Y (1992). Centromere protein B assembles human centromeric alpha-satellite DNA at the 17-bp sequence, CENP-B box. J Cell Biol.

[40] Wong LH (2007). Centromere RNA is a key component for the assembly of nucleoproteins at the nucleolus and centromere. Genome Res.

[41] Moyzis RK (1987). Human chromosome-specific repetitive DNA sequences: novel markers for genetic analysis. Chromosoma.

[42] Shih HM, Chang CC, Kuo HY, Lin DY (2007). Daxx mediates SUMO-dependent transcriptional control and subnuclear compartmentalization. Biochem Soc Trans.

[43] Sahin U (2014). Oxidative stress-induced assembly of PML nuclear bodies controls sumoylation of partner proteins. J Cell Biol.

[44] Lin DY (2006). Role of SUMO-interacting motif in Daxx SUMO modification, subnuclear localization, and repression of sumoylated transcription factors. Mol Cell.

[45] Santiago A, Godsey AC, Hossain J, Zhao LY, Liao D (2009). Identification of two independent SUMO-interacting motifs in Daxx: evolutionary conservation from Drosophila to humans and their biochemical functions. Cell Cycle.

[46] Tatham MH (2001). Polymeric chains of SUMO-2 and SUMO-3 are conjugated to protein substrates by SAE1/SAE2 and Ubc9. J Biol Chem.

[47] Geiss-Friedlander R, Melchior F (2007). Concepts in sumoylation: a decade on. Nat Rev Mol Cell Biol.

[48] Hickey CM, Wilson NR, Hochstrasser M (2012). Function and regulation of SUMO proteases. Nat Rev Mol Cell Biol.

[49] Tatham MH (2008). RNF4 is a poly-SUMO-specific E3 ubiquitin ligase required for arsenic-induced PML degradation. Nat Cell Biol.

[50] Lallemand-Breitenbach V (2008). Arsenic degrades PML or PML-RARalpha through a SUMO-triggered RNF4/ubiquitin-mediated pathway. Nat Cell Biol.

[51] Gisselsson D (2008). Classification of chromosome segregation errors in cancer. Chromosoma.

[52] Guenatri M, Bailly D, Maison C, Almouzni G (2004). Mouse centric and pericentric satellite repeats form distinct functional heterochromatin. J Cell Biol.

[53] Vagnarelli P, Ribeiro SA, Earnshaw WC (2008). Centromeres: old tales and new tools. FEBS Lett.

[54] Jang CW, Shibata Y, Starmer J, Yee D, Magnuson T (2015). Histone H3.3 maintains genome integrity during mammalian development. Genes Dev.

[55] Baumann C, Viveiros MM, De La Fuente R (2010). Loss of maternal ATRX results in centromere instability and aneuploidy in the mammalian oocyte and pre-implantation embryo. PLoS Genet.

[56] De La Fuente R, Baumann C, Viveiros MM (2011). Role of ATRX in chromatin structure and function: implications for chromosome instability and human disease. Reproduction.

[57] Lovejoy CA (2012). Loss of ATRX, genome instability, and an altered DNA damage response are hallmarks of the alternative lengthening of telomeres pathway. PLoS Genet.

[58] Marinoni I, et al. (2014) Loss of DAXX and ATRX are associated with chromosome instability and reduced survival of patients with pancreatic neuroendocrine tumors. *Gastroenterology* 146(2):453–460 e455. 10.1053/j.gastro.2013.10.020 24148618

[59] Ribeiro SA (2010). A super-resolution map of the vertebrate kinetochore. Proc Natl Acad Sci USA.

[60] Rapkin LM (2015). The histone chaperone DAXX maintains the structural organization of heterochromatin domains. Epigenetics Chromatin.

[61] Gerlich D, Hirota T, Koch B, Peters JM, Ellenberg J (2006). Condensin I stabilizes chromosomes mechanically through a dynamic interaction in live cells. Curr Biol.

[62] Ribeiro SA (2009). Condensin regulates the stiffness of vertebrate centromeres. Mol Biol Cell.

[63] Nasmyth K, Haering CH (2009). Cohesin: its roles and mechanisms. Annu Rev Genet.

[64] Hudson DF (1998). Centromere protein B null mice are mitotically and meiotically normal but have lower body and testis weights. J Cell Biol.

[65] Kapoor M (1998). The cenpB gene is not essential in mice. Chromosoma.

[66] Perez-Castro AV (1998). Centromeric protein B null mice are viable with no apparent abnormalities. Dev Biol.

[67] Grewal SI (2010). RNAi-dependent formation of heterochromatin and its diverse functions. Curr Opin Genet Dev.

[68] Dunleavy EM, Almouzni G, Karpen GH (2011). H3.3 is deposited at centromeres in S phase as a placeholder for newly assembled CENP-A in G(1) phase. Nucleus.

[69] Maison C (2011). SUMOylation promotes de novo targeting of HP1alpha to pericentric heterochromatin. Nat Genet.

[70] Muchardt C (2002). Coordinated methyl and RNA binding is required for heterochromatin localization of mammalian HP1alpha. EMBO Rep.

[71] Maison C (2002). Higher-order structure in pericentric heterochromatin involves a distinct pattern of histone modification and an RNA component. Nat Genet.

[72] Udugama M (2015). Histone variant H3.3 provides the heterochromatic H3 lysine 9 tri-methylation mark at telomeres. Nucleic Acids Res.

[73] Elsasser SJ, Noh KM, Diaz N, Allis CD, Banaszynski LA (2015). Histone H3.3 is required for endogenous retroviral element silencing in embryonic stem cells. Nature.

[74] Kimura A, Horikoshi M (2004). Partition of distinct chromosomal regions: negotiable border and fixed border. Genes Cells.

[75] Flotho A, Melchior F (2013). Sumoylation: a regulatory protein modification in health and disease. Annu Rev Biochem.

[76] Citro S, Chiocca S (2013). Sumo paralogs: redundancy and divergencies. Front Biosci.

[77] Saitoh H, Hinchey J (2000). Functional heterogeneity of small ubiquitin-related protein modifiers SUMO-1 versus SUMO-2/3. J Biol Chem.

[78] Kolli N (2010). Distribution and paralogue specificity of mammalian deSUMOylating enzymes. Biochem J.

[79] Schimmel J (2014). Uncovering SUMOylation dynamics during cell-cycle progression reveals FoxM1 as a key mitotic SUMO target protein. Mol Cell.

[80] Golebiowski F (2009). System-wide changes to SUMO modifications in response to heat shock. Sci Signal.

[81] Tatham MH, Matic I, Mann M, Hay RT (2011). Comparative proteomic analysis identifies a role for SUMO in protein quality control. Sci Signal.

[82] Ray-Gallet D (2011). Dynamics of histone H3 deposition in vivo reveal a nucleosome gap-filling mechanism for H3.3 to maintain chromatin integrity. Mol Cell.

[83] Xue Y (2003). The ATRX syndrome protein forms a chromatin-remodeling complex with Daxx and localizes in promyelocytic leukemia nuclear bodies. Proc Natl Acad Sci USA.

[84] McDowell TL (1999). Localization of a putative transcriptional regulator (ATRX) at pericentromeric heterochromatin and the short arms of acrocentric chromosomes. Proc Natl Acad Sci USA.

[85] Iwase S (2011). ATRX ADD domain links an atypical histone methylation recognition mechanism to human mental-retardation syndrome. Nat Struct Mol Biol.

[86] Law MJ (2010). ATR-X syndrome protein targets tandem repeats and influences allele-specific expression in a size-dependent manner. Cell.

[87] Escobar-Cabrera E (2011). Characterizing the N- and C-terminal Small ubiquitin-like modifier (SUMO)-interacting motifs of the scaffold protein DAXX. J Biol Chem.

[88] Chang CC (2011). Structural and functional roles of Daxx SIM phosphorylation in SUMO paralog-selective binding and apoptosis modulation. Mol Cell.

[89] Nakatani Y, Ogryzko V (2003). Immunoaffinity purification of mammalian protein complexes. Methods Enzymol.

[90] Boddy MN, Howe K, Etkin LD, Solomon E, Freemont PS (1996). PIC 1, a novel ubiquitin-like protein which interacts with the PML component of a multiprotein complex that is disrupted in acute promyelocytic leukaemia. Oncogene.

[91] Shalem O (2014). Genome-scale CRISPR-Cas9 knockout screening in human cells. Science.

