# Supplementary figures and images for: CENP-B protects centromere chromatin integrity by facilitating histone deposition via the H3.3-specific chaperone Daxx
Source: Epigenetics Chromatin. 2017 Dec 22;10:63. doi: 10.1186/s13072-017-0164-y (PMC5741900; doi:10.1186/s13072-017-0164-y)

**A**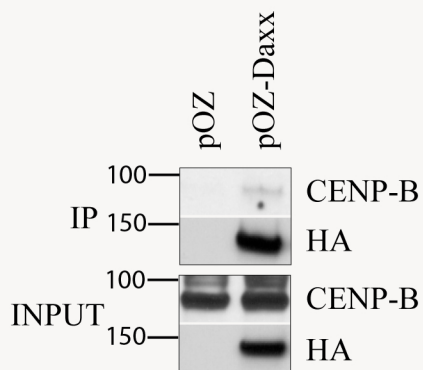**B**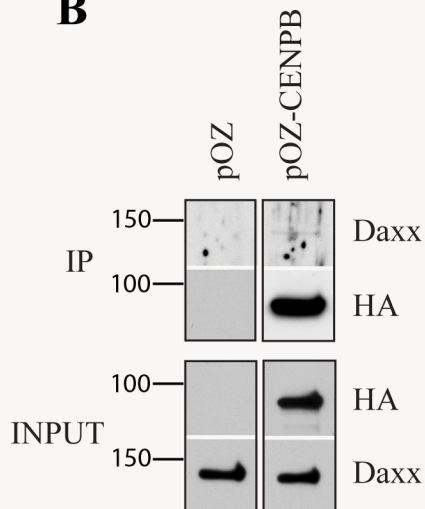

Supplement: Supplementary file 1 — Additional file 1: Fig. S1. Association of CENP-B and Daxx in chromatin fraction (relevant to Fig. 1a). A. FLAG IP from chromatin fraction of HEp2 cells expressing FLAG-HA-Daxx probed with CENP-B antibodies. B. Reciprocal IP from chromatin fraction of HEp2 cells expressing FLAG-HA-CENP-B probed with Daxx antibodies. [file 13072_2017_164_MOESM1_ESM.pdf]

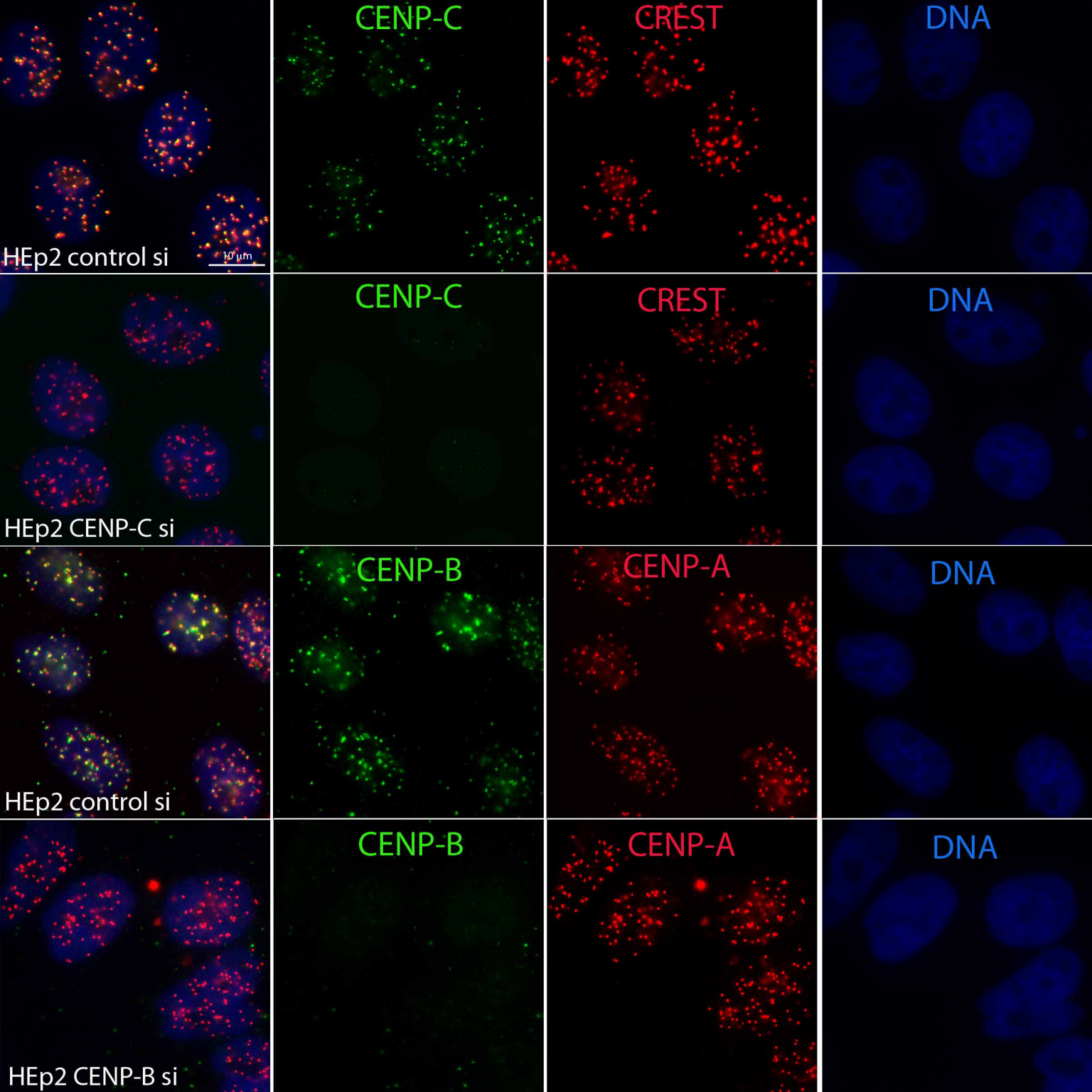

Morozov et al., Fig. S2

Supplement: Supplementary file 2 — Additional file 2: Fig. S2. CENP-B and CENP-C depletion efficiency (relevant to Fig. 1b). Representative images of HEp2 cells transfected with scrambled (control), CENP-B and -C siRNA and stained for CENP-C (green) and CREST (red) (top and second rows) or CENP-B (green) and CENP-A (red) (third and bottom rows); DNA blue. Both siRNAs significantly reduced levels of corresponding centromere proteins. [file 13072_2017_164_MOESM2_ESM.pdf]

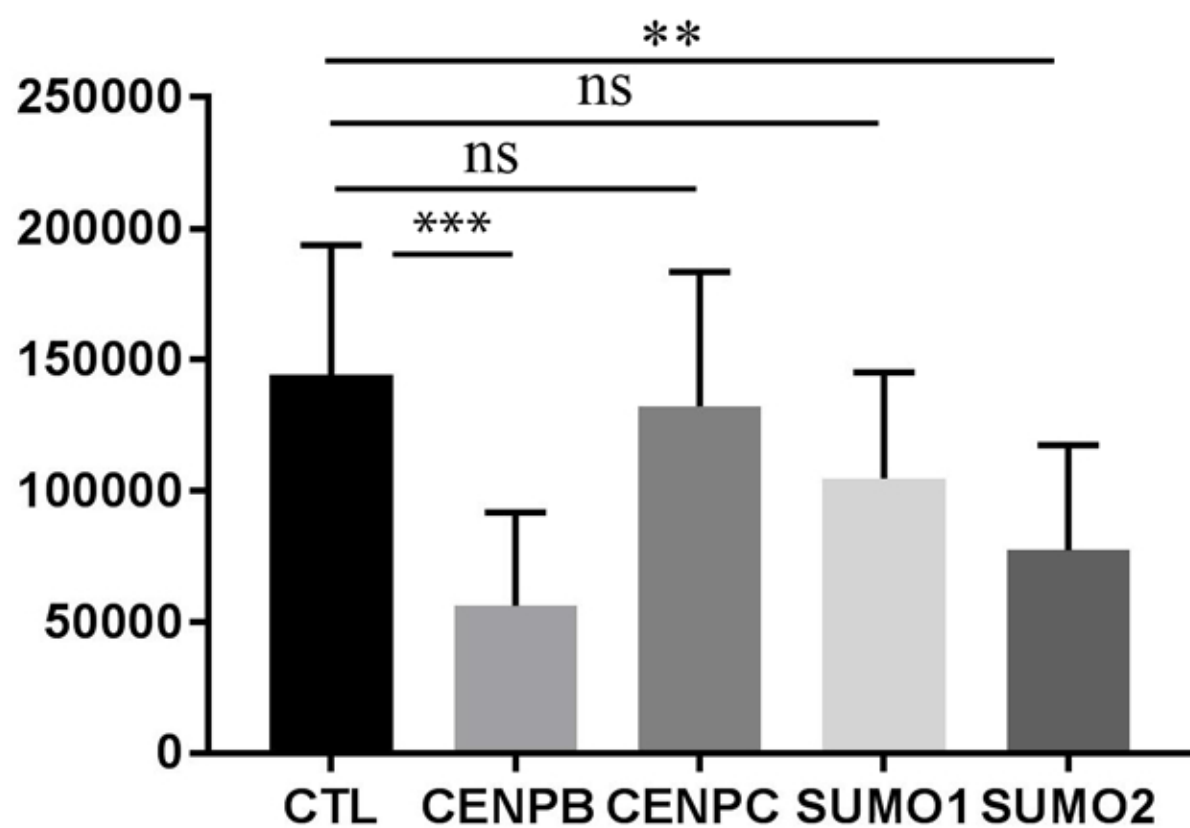

Supplement: Supplementary file 3 — Additional file 3: Fig. S3. Quantitative analysis of Daxx intensity staining at centromeres (relevant to Figs. 1b and 4a). Data in arbitrary units for control-, CENP-B-, CENP-C-, SUMO-1-, and SUMO-2-depleted cells after MG132 treatment of HEp2 cells. Statistical analysis was performed by one-way ANOVA followed by Dunnett’s multiple comparisons test. The bars represent mean ± SD. P < 0.001: ***; P < 0. 01: **; NS: nonsignificant. [file 13072_2017_164_MOESM3_ESM.pdf]

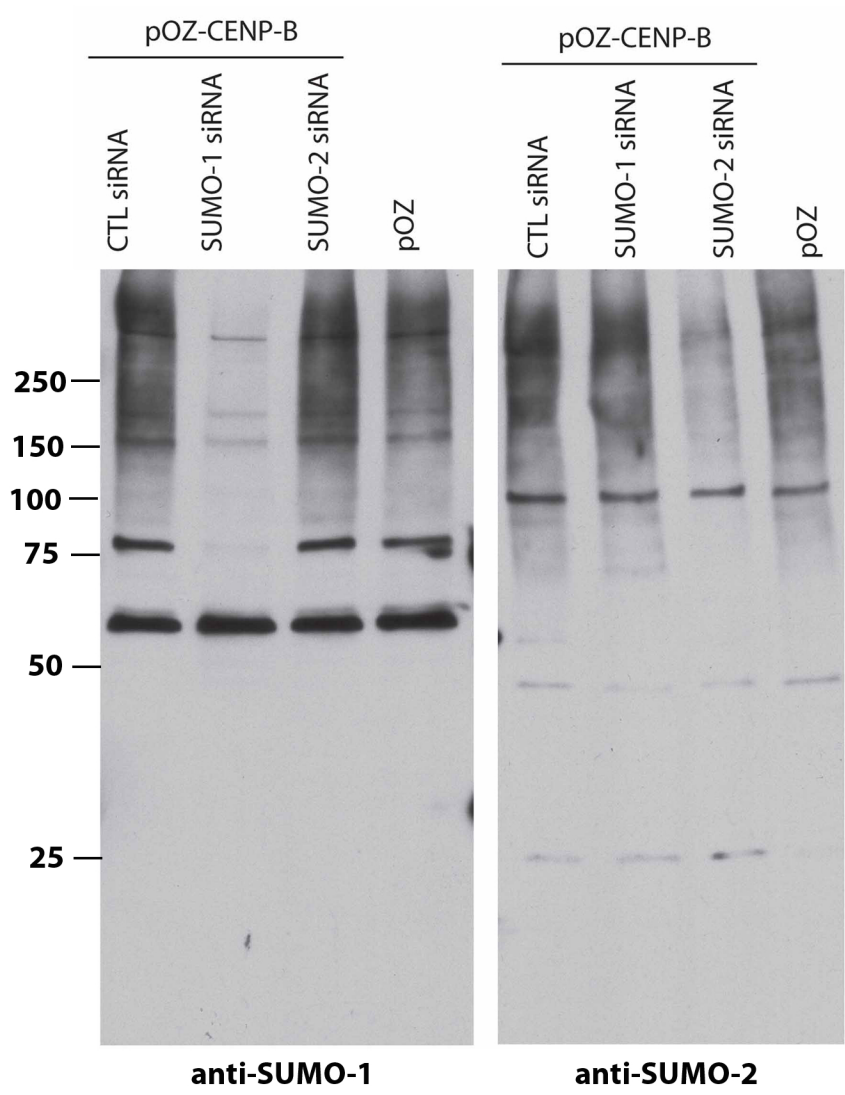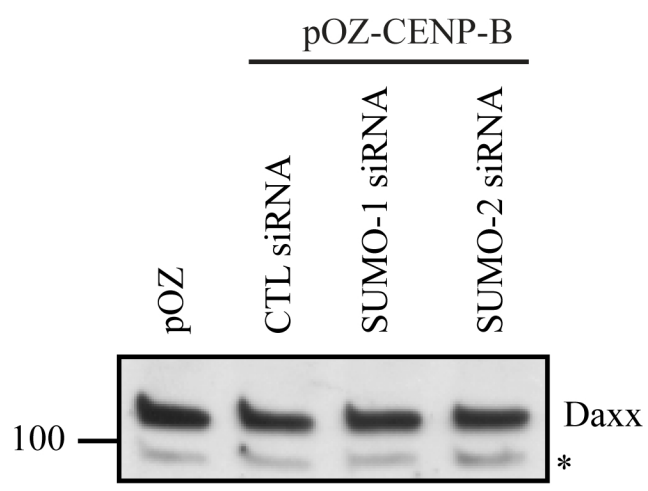

Supplement: Supplementary file 4 — Additional file 4: Fig. S4. Top: SUMO depletion efficiency (relevant to Fig. 4). HEp2 cells expressing FLAG-HA-CENP-B (pOZ-CENP-B) were transfected with scrambled siRNA (CTL), SUMO-1 or -2 siRNA and treated with MG132 for 4 h. Cell lysates were analyzed by Western blot and probed with SUMO-1 (left) or SUMO-2 (right) antibodies. Control: un-transfected HEp2 cells (pOZ). Both SUMO siRNA reduced levels of corresponding conjugates. Bottom: SUMO depletion does not change levels of endogenous Daxx (relevant to Fig. 3c). Conditions as above; chromatin-associated fractions were analyzed by Western blot and probed with Daxx antibodies. *: unspecific band. Control: un-transfected HEp2 cells (pOZ). SUMO-1 or -2 depletion does not affect levels of Daxx. [file 13072_2017_164_MOESM4_ESM.pdf]

A

MCF7

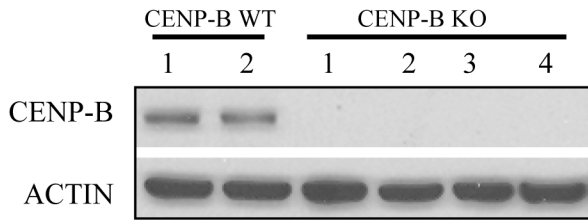

HEp2

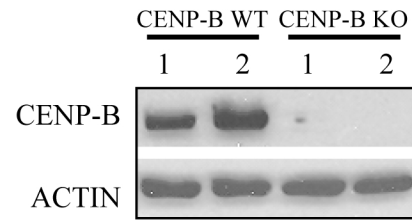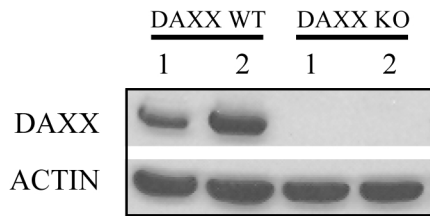

B

% micronuclei

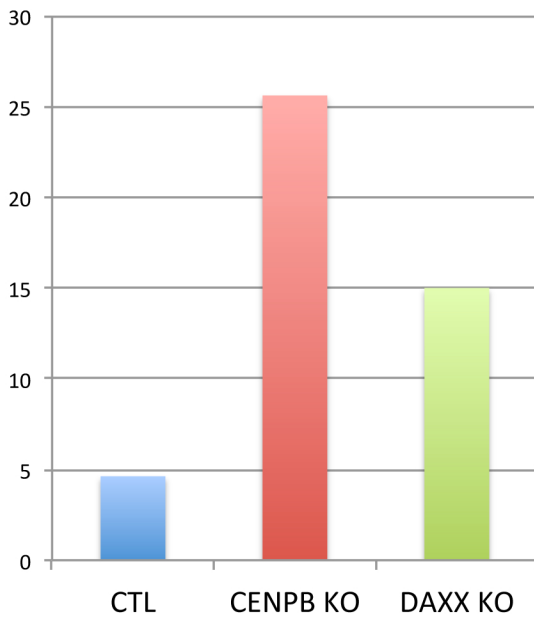

% lagging chromosomes

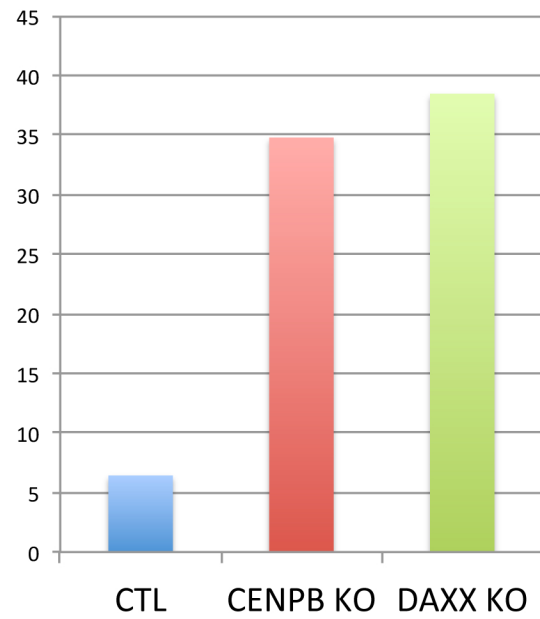

Supplement: Supplementary file 5 — Additional file 5: Fig. S5. A. Western blot analysis of HEp2 and MCF7 CENP-B and Daxx knockout clones. B. Analysis of micronuclei and lagging chromosomes in MCF-7 knockout clones. At least 100 mitotic events were analyzed for each clone. [file 13072_2017_164_MOESM5_ESM.pdf]

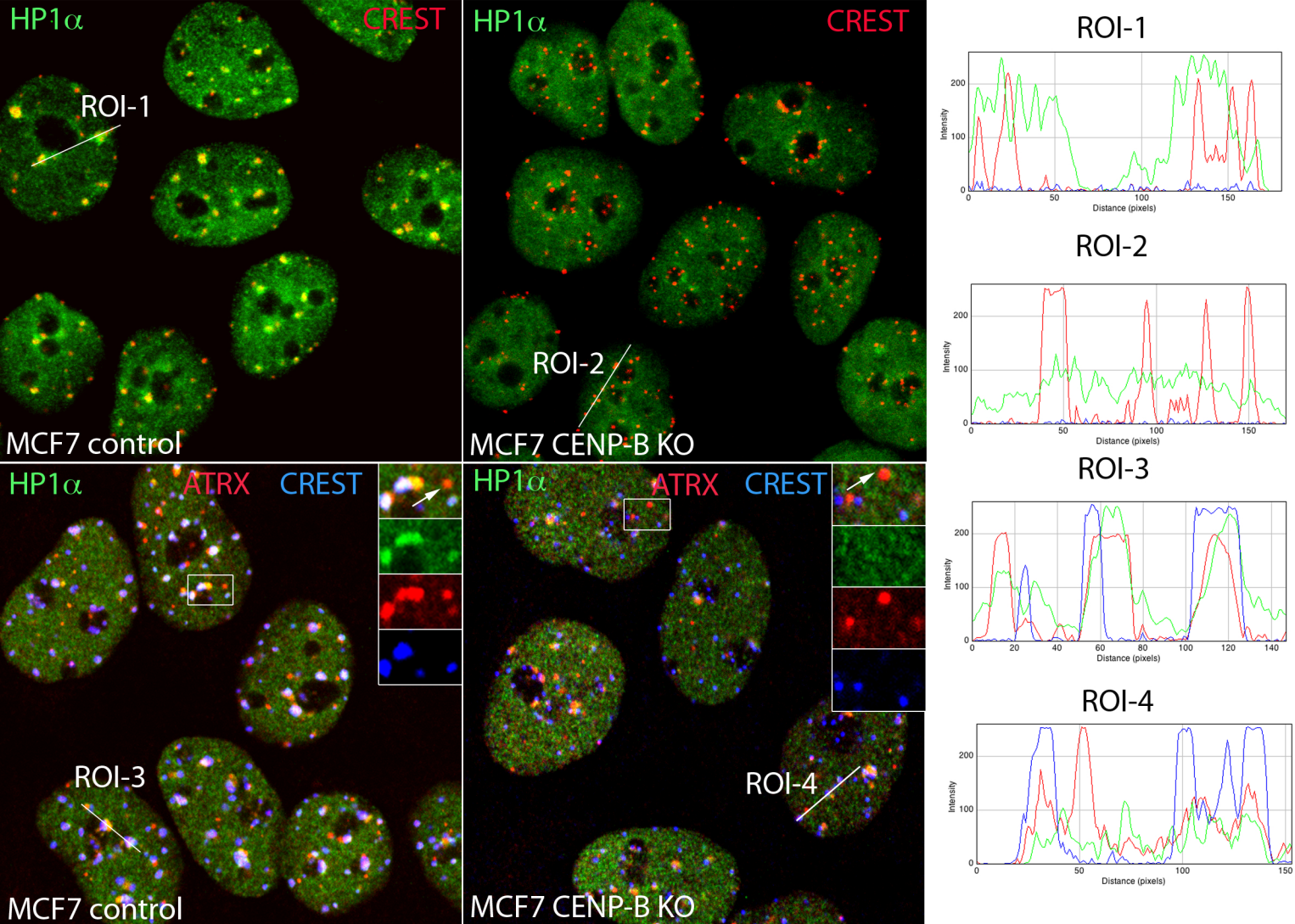

Morozov et al., Fig. S6

Supplement: Supplementary file 6 — Additional file 6: Fig. S6. CENP-B and Daxx knockout reduce HP1α accumulation at centromeres. Representative images of MCF7 control (left column) and CENP-B knockout (right column). Top row and ROI-1/-2: knockout of CENP-B (right) reduced accumulation of key heterochromatin protein HP1α (green) at centromeres (CREST, blue). Bottom row and ROI-4: knockout of CENP-B (right) reduced co-localization of HP1α (green) and ATRX (red) at centromeres (CREST, blue). PML nuclear bodies marked with arrows. [file 13072_2017_164_MOESM6_ESM.pdf]
